# Unravelling Secondary Brain Injury: Insights from a Human-Sized Porcine Model of Acute Subdural Haematoma

**DOI:** 10.3390/cells14010017

**Published:** 2024-12-27

**Authors:** Thomas Kapapa, Vanida Wernheimer, Andrea Hoffmann, Tamara Merz, Fabia Zink, Eva-Maria Wolfschmitt, Oscar McCook, Josef Vogt, Martin Wepler, David Alexander Christian Messerer, Claire Hartmann, Angelika Scheuerle, René Mathieu, Simon Mayer, Michael Gröger, Nicole Denoix, Enrico Clazia, Peter Radermacher, Stefan Röhrer, Thomas Datzmann

**Affiliations:** 1 Department of Neurosurgery, University Hospital Ulm, Albert-Einstein-Allee 23, 89081 Ulm, Germany; 2 Institute of Anaesthesiologic Pathophysiology and Process Development, University Hospital Ulm, Helmholtzstrasse 8/1, 89081 Ulm, Germany; 3 Department of Anaesthesiology, University Hospital Ulm, Albert-Einstein-Allee 23, 89081 Ulm, Germany; 4 Institute of Transfusion Medicine, University Hospital Ulm, Helmholtzstrasse 10, 89081 Ulm, Germany; 5 Section Neuropathology, University Hospital Ulm, Albert-Einstein-Allee 23, 89081 Ulm, Germany; 6 Department of Neurosurgery, Military Hospital Ulm, Oberer Eselsberg 40, 89081 Ulm, Germany; 7 Department of Neurosurgery, Ostalb-Hospital Aalen, Im Kälblesrain 1, 73430 Aalen, Germany

**Keywords:** secondary brain injury, animal model, acute subdural haematoma, traumatic brain injury, management, guidelines, multimodal brain monitoring, outcome, intracranial pressure

## Abstract

Traumatic brain injury (TBI) remains one of the leading causes of death. Because of the individual nature of the trauma (brain, circumstances and forces), humans experience individual TBIs. This makes it difficult to generalise therapies. Clinical management issues such as whether intracranial pressure (ICP), cerebral perfusion pressure (CPP) or decompressive craniectomy improve patient outcome remain partly unanswered. Experimental drug approaches for the treatment of secondary brain injury (SBI) have not found clinical application. The complex, cellular and molecular pathways of SBI remain incompletely understood, and there are insufficient experimental (animal) models that reflect the pathophysiology of human TBI to develop translational therapeutic approaches. Therefore, we investigated different injury patterns after acute subdural hematoma (ASDH) as TBI in a post-hoc approach to assess the impact on SBI in a long-term, human-sized porcine TBI animal model. Post-mortem brain tissue analysis, after ASDH, bilateral ICP, CPP, cerebral oxygenation and temperature monitoring, and biomarker analysis were performed. Extracerebral, intraparenchymal–extraventricular and intraventricular blood, combined with brainstem and basal ganglia injury, influenced the experiment and its outcome. Basal ganglia injury affects the duration of the experiment. Recognition of these different injury patterns is important for translational interpretation of results in this animal model of SBI after TBI.

## 1. Introduction

Traumatic brain injury (TBI) is one of the most common human injuries, occurring approximately 27 to 69 million times per year worldwide, with a prevalence of approximately 49 million cases and 7.1 million years lived with disability (YLD) [1,2,3]. It is also one of the most common diseases of the central nervous system [3,4]. In Europe and the USA, TBI was the cause of 37% of deaths following injury in 2012 and the number of deaths from TBI was estimated at it was 82,000 people or 17.5 TBI-related deaths per 100,000 inhabitants in the years 2000 and 2005 [5,6]. The direct and indirect costs for the care of patients after TBI are estimated internationally to be in the triple-digit billion range [7,8]. These figures show that TBI research has a significant individual, social and economic impact on par with other diseases.

The causes of a TBI show regional differences with an accumulation of young victims from traffic accident situations in low-income countries (LICs), especially in the global South, compared to older patients after falls in middle- to high-income countries, especially in the global North [1,4]. In many countries, the leading cause of TBI is falls (74%), followed by pedestrian road injuries (14%), motor vehicle road injuries (5%) and conflict and terrorism victims (2%) [1]. Although older chronological age is often associated with poorer outcomes, there are no guidelines for TBI in the elderly. [4,9]. Unfortunately, the recommendations in the guidelines for TBI in childhood can only rarely be attributed to a higher level of evidence (Level I) [10,11,12]. The influence of social or biological age compared to chronological age on the outcome after TBI is currently the subject of various clinical studies focussing on frailty and comorbidities [9]. This means that especially at the two vulnerable ends of the chronological view of life (children and older adults), the evidence base for clinical management is insufficient to make high-level evidence recommendations.

TBI can be categorised differently from a clinical (Glasgow Coma Scale), diagnostic (Imaging), aetiology (blunt, penetrating, blast), area of involvement (diffuse, focal) and pathophysiological (primary or secondary) point of view [13,14]. Starting from the premise that consciousness is one of the most important and highest functions of the brain, in 1974, Jennett and Teasdale attempted to categorise the impairment of brain function after TBI on the basis of consciousness [15]. Using the variables of best eye opening, best motor response and best verbal response, they created a practical scale that is still used today to differentiate between mild (15–13 points), moderate (12–9 points) and severe (≤9 points) TBI: the Glasgow Coma Scale (GCS) [15,16,17]. From a value of 9 to 3, coma becomes increasingly likely [15,18]. However, the GCS has only limited predictive capabilities with regard to outcome [19,20]. The majority of TBI patients treated in hospital have a mild TBI (90%) [4]. The remaining 10% are moderate and severe TBIs. Moderate, and especially severe TBI, have a very high mortality rate of up to 30% [21,22]. The majority of patients receive a computed tomography (CT) scan to clarify their brain injury, which often shows no structural damage in mild TBI [4]. However, the absence of structural damage on a CT does not mean that there is no damage. Magnetic resonance imaging (MRI) can in some cases reveal damage that is not visible on CT. This is consistent with the fact that many patients with mild TBI, as measured by the GCS, for example, do not return to their pre-injury level of performance [4]. Different scoring systems exist to categorise the severity of TBI using imaging, corresponding to the different modalities of CT [23,24,25,26] and MRI [27]. Image diagnosis distinguishes between extracranial injuries (skin lacerations and subgaleal haemorrhages), cranial injuries (fractures and suture splinters), extracerebral–epidural injuries (epidural haematomas), extracerebral-intradural injuries (acute and chronic subdural haemorrhage, subarachnoid haemorrhage), intracerebral haemorrhage (traumatic intracerebral haemorrhage and contusion), intraventricular haemorrhage and diffuse injuries and combinations [14,28,29,30,31,32,33,34,35,36,37]. For example, the score according to Marshall et al. [38] distinguishes between the absence of intracranial pathology, compression of the basal cisterns, midline shift or the size of traumatically induced lesions such as contusions. The score according to Maas et al. [24] distinguishes between compression of the basal cisterns, midline shift, the presence of traumatic epidural space-occupying lesions (extradural), intraventricular blood (intracerebral-intraventricular) or traumatic subarachnoid haemorrhage (intradural-extracerebral). Firsching et al. differentiate in MRI between purely supratentorial injuries versus unilateral and bilateral injuries anywhere at the brain stem, the mesencephalon or the pons [27]. All scores show that a higher degree and involvement of the brain stem is associated with a poorer outcome or even a higher probability of death [38,39,40]. Thus, the gradations of consciousness and the different patterns of injury play an important role in clinical severity classification, treatment planning and prediction of functional outcome.

Pathophysiologically, TBI can be divided into primary (early brain injury, EBI), which represents the direct effect of force on the brain and its linings, including fractures and haemorrhages, and secondary (delayed brain injury, DBI), which represents all the cellular and molecular consequences, including the formation of oedema, ischaemia, seizures and spreading depolarisation [41,42,43]. During the primary trauma, the brain and its surroundings are subjected to a force that may be direct focal, rotational or shearing. These forces do not usually occur separately, but together to varying degrees. These forces act on an individual brain. For example, rotational forces can lead to axonal rupture in the white matter, resulting in diffuse axonal damage reaching into the basal ganglia or the brainstem [43,44]. This makes each TBI individual. Focal injuries of the primary trauma can occur directly (e.g., fractures, vascular laceration under the fracture with epidural, subdural or intracerebral haematoma) or indirectly, as a result of acceleration and deceleration of the brain (contusion against the directly acting force), and lead to increased ICP [44]. The consequences of the primary trauma can be subclinical, clinically mild, moderate or severe and can manifest themselves in initial unconsciousness, reduced alertness and impaired ability to react and communicate, right through to unaffectedness [43]. Secondary trauma immediately follows primary trauma and includes processes and events such as blood-brain-barrier (BBB) breakdown, excitotoxicity, mitochondrial dysfunction, oxidative stress, neuroinflammation, axonal degeneration and apoptotic cell death [45,46]. In neurons, stretching or rupture of the cell membrane leads to disruption of sodium (Na^+^) influx, potassium (K^+^) efflux and an increase in intra-axonal calcium (Ca2^+^). This activates proteases, calpain and calpain-mediated proteolysis of the cytoskeleton [47,48,49]. The result is irreversible axonal damage [49,50]. Neurones undergoing apoptosis and necrosis release masses of damage-associated molecular patterns (DAMPs: extravascular vesicles, microparticles, fibrinogen, annexins, platelet components, fibronectin, S100 proteins, syndecan-1, F-actin, adenosine-5-triphosphate, histone, DNA, high mobility group box protein-1) which in turn activate resident inflammatory cells (microglia and t-cells) to release cytokines [42,51,52]. Further Ca2^+^ influx leads to neuronal depolarisation and increased glucose consumption. Cellular energy reserves are rapidly depleted, leading to mitochondrial dysfunction with impaired oxidative metabolism and lactate production. The end result of this abbreviated cascade is acidosis and cerebral oedema [49,53,54]. When the described damage impacts endothelial cells, pericytes, astrocytes (with their foot processes) and the basement membrane, which we understand as a coherent structure called the BBB, the damage to neurons is even greater due to the disturbed homeostasis [55,56]. Already ongoing inflammatory processes, partly fuelled by microglia (IL-1β, IFNγ, TNFα, nitric oxide, ROS), are exacerbated by the uncontrolled influx of immunologically and inflammationally active substances, such as chemokines, cytokines and cells of the peripheral blood circulation (macrophages, neutrophils, t- and b-lymphocytes), as well as by the breakdown of the selective permeability function of tight and adherens junctions in the BBB (impaired claudin-5 function) [42,47,51,57,58,59,60,61]. The constantly increasing extracellular oncotic pressure (impaired endothelial cell aquaporin 1 and 4 function) leads to a further increase in ICP with herniation and midline shift, and thus to a reduction in cerebral perfusion pressure (CPP), which is a surrogate for cerebral blood flow (CBF), and an increase in ischaemia [59,62,63]. The disruption of astrocyte homeostasis (uptake of albumin via the TGF^ᵝ^ receptor) leads to further structural damage to the brain [47]. Cytotoxic cerebral oedema (loss of Na^+^/K^+^-ATPase and Ca^2+^-ATPase, ion channels and cotransporters (NKCC1) and Na^+^/Ca^2+^ exchange lead to impaired membrane transport and fluid accumulation in cells) parallels vasogenic cerebral oedema [64,65]. These processes, some of which occur in parallel, usually reach their maximum 3–4 days after the trauma and lead to brain death in a vicious circle that cannot be broken by surgical measures [66]. This means that consideration of the clinical and pathophysiological consequences of TBI extends over a period of several days after the trauma and that various therapeutic approaches to the treatment of secondary trauma are obscured during this period. The term TBI therefore represents a heterogeneous collection of injuries.

Surgical treatment and neurointensive care of TBI, and especially severe TBI with different injury patterns, is subject to guidelines [67,68,69]. These include recommendations on the use of hyperosmolar substance administration for the non-surgical treatment of cerebral oedema and increased ICP, analgesia, further monitoring and treatment of ICP, blood pressure and CPP, as well as surgical measures such as decompressive craniectomy (DC). However, the studies and data available to date have not shown a significant breakthrough in demonstrating an advantage of non-surgical over surgical treatment approaches in improving the functional outcome of patients [8,60,70,71,72,73,74,75,76,77,78,79,80]. DC, as removing large parts of the supratentorial skull, is able to reduce therapy-resistant ICP, but does not significantly improve functional outcome, which is similar to ICP-guided therapy [67,81,82,83,84,85,86,87]. There are significant gaps in the knowledge of the pathophysiology in different injury patterns that do not always allow clinical practice to achieve a very good functional outcome.

Even after several decades of intensive research, more than 28,000 publications and more than 250 trials, there are unfortunately no effective drug approaches for the effective treatment of severe TBI that improve the survival and functional outcome [22,88]. The reasons for this were assumed to be ‘Failure of methodological rigour in preclinical research to ensure experimental reproducibility and high-quality data for human translation’, ‘poorly designed human trials’, ‘Failure of animal models to replicate the human condition’ or ‘Flawed practice of single-nodal targeting in preclinical research’ [22]. Numerous studies discuss the ongoing problem of translation from animals to humans in TBI research [88,89,90,91,92,93,94]. In addition, the lack of highly funded and holistic scientific research (clinical and basic research) in favour of single-nodal targeting research is seen as the cause of a lack of translation [22]. Focusing on one step, one particular pathway, agent, symptom or treatment strategy to understand the processes of secondary brain damage has not yet led to success [95,96,97,98]. This focussed strategy is not generally regarded as wrong, but it did not lead to significant successes for TBI [22]. There are numerous approaches using animal experiments to investigate the controversies of ICP monitoring, DC and, above all, TBI in childhood. However, these approaches rarely include longer phases of intensive medical therapy (as in humans) for long-term investigation of secondary brain damage (4 to 17 days), a trauma mechanism (e.g., traffic accidents with different vectors of forces) and neuromonitoring (as in humans) or take into account extra-cranial complications or comorbidities (pulmonary, cardiac, coagulation, haemorrhagic shock), age and sex (focused research approach) [99,100,101,102,103,104,105,106,107,108,109,110,111,112,113].

In our present study, we analysed the impact of different injury patterns, as mentioned above, on secondary brain injury after acute subdural haematoma (ASDH) in a longtime porcine animal model, including neurocritical care measures. ASDH is a common finding in TBI (49%) after falls and traffic accidents, particularly in the elderly and those with comorbidities [114,115], and represents one of the most serious consequences of TBI, as it is associated with high mortality (30–67%) and morbidity (19–62%) [116,117,118,119,120,121,122,123,124]. ASDH can result in increased ICP, reduced CPP, regional or global ischemia, and the formation of cytotoxic or vasogenic oedema [69,118,125,126,127,128,129]. The therapeutic approach that leads to improved functional outcome (non-surgical versus decompressive craniectomy) is the subject of much debate [76,124,130,131,132]. Specifically, based on the immediate postmortem macroscopic aspect, we sought to differentiate the role of the location of the intracranial blood distribution as well as the involvement of the brainstem and/or basal ganglia for secondary brain injury. The data presented are a post-hoc analysis of material available from different previous studies characterising the model as well as using this model in combination with haemorrhagic shock [133,134,135].

The aim of this analysis is to determine the influence of different injury patterns and their secondary trauma on the post-traumatic survival time in the experiment, on the respective impairments of consciousness (a modified GCS) and on the vital and neuromonitoring parameters, as well as on the anaesthesia parameters and biomarkers within this long-term intensive-care large animal model for TBI.

## 2. Materials and Methods

All experiments have been conducted in adherence with the National Institutes of Health Guidelines on the Use of Laboratory Animals and the European Union “Directive 2010/63/EU on the protection of animals used for scientific purposes” and were performed after obtaining approval from the local Animal Care Committee and the Federal Authorities for Animal Research (Regierungspräsidium Tübingen, Germany, #1316, approval dated 2 May 2017) [133,134,135].

### 2.1. Animal Model

Since in 4 animals the experiments had to be terminated prematurely due to surgical, neuro-biological or cardio-vascular complications (5, 8, 15 and 26), the present data report on the analysis of 44 animals (26 males, 22 females), consisting of 24 Bretoncelles–Meishan–Willebrand (BMW) pigs (mean age 17 months (SD: 2); 16 castrated males, mean body weight 71 kg (SD: 8); 8 females, mean body weight 72 kg (SD: 5)) and 20 Familial hypercholesterolemia, Bretoncelles and Meishan (FBM) pigs (mean age 38 months (SD: 9); 9 castrated males, mean body weight 66 kg (SD: 12); 11 females, mean body weight 73 kg (SD: 15)). The otherwise healthy BMW strain presents with a reduced activity of the von Willebrand Factor (vWF), in contrast to the hypercoagulatory state in domestic swine strains [136], thereby mimicking human coagulation activity [137]. Due to a genetic mutation and an atherogenic diet, the FBM strain is characterised by ubiquitous arteriosclerosis and consecutive coronary artery disease that resembles that of a human with a cardiac comorbidity and therefore is close to the experimental design in (older) premorbid patients [138,139].

### 2.2. Composition of the Test Animal Groups and Approach of the Post-Hoc Analysis

This manuscript represents the post-hoc analysis of previous experimental series. These involved the characterisation of the ASDH model itself [133,134,135] and the simulation of blood loss (haemorrhagic shock N = 34, 77.3%) [134,135]. The blood loss intervention represents passive removal of 30% of the calculated total blood amount over 30 min as a simulation of additional extracranial injury [134]. Case estimates and power analyses were performed for each of these individual experiments.

The reason why both groups of animals (with and without additional blood loss) were pooled and analysed together is that they have the same primary brain damage. The shock situation later in the experiment (secondary phase) should have no effect on the primary damage. This is useful for differentiation of whether the injury patterns and distribution of the haemorrhage are the result of primary or secondary injury. Further, this allowed the number of animals used to analyse primary trauma, injury patterns and blood distribution to be increased.

The ‘3R’ principle was taken into account by implanting bi-hemispheric monitoring catheters; in other words, the hemisphere without ASDH was the control for the hemisphere with ASDH. This made it possible to avoid sham-operated animals.

### 2.3. Experimental Protocol

The anaesthesia and (neuro-)surgical instrumentation and interventions have been described in detail previously [133,134,135,140]. Briefly, prior to the experiment, animals had free access to water and were fed with caloric food (Fresubin, Fresenius Kabi, Bad Homburg von der Höhe, Germany). Premedication comprised i.m. application of azaperone (5 mg/kg) and 1–2 mg/kg midazolam. After establishment of a venous access via an ear vein, anaesthesia was initiated with propofol (1–2 mg/kg) and ketamine (1 mg/kg) followed by endotracheal intubation and controlled mechanical ventilation (tidal volume 8 mL/kg respiratory rate 8–12 breaths/minute) to achieve an arterial PCO_2_ (PaCO_2_) of 35–40 mmHg, inspiration-to-expiration ratio (I/E ratio) of 1:1.5, inspiratory O_2_ concentration (F_I_O_2_) of 30% and positive end-expiratory pressure (PEEP) of 10 cm H_2_O. Anaesthesia was maintained by continuous i.v. propofol (10 mg / kg / hour) and fentanyl (10 μg/kg initially and then 2.5 μg/kg) [133] or remifentanil (5 µg/kg initial bolus, followed by 15–20 µg/kg and per hour) [134,135]. Maintenance fluid administration (10 mL/kg × h) was a balanced electrolyte solution (Jonosteril 1/1, Fresenius Kabi, Bad Homburg von der Höhe, Germany) [141]. A 3-lumen catheter was surgically inserted into the left femoral artery (9-French, Arrow International Inc.,Reading, Pennsylvania (PA), USA), a 4-French PiCCO catheter was inserted into the right superficial femoral artery for continuous blood pressure and cardiac output monitoring and a urinary catheter was inserted via a mini-laparotomy [133].

Neurosurgical instrumentation was performed through a midline incision via the frontal and parietal skull-bone. A burr hole (approx. 10 mm) was drilled bilaterally using an electric trepan and surgical fraise (ELAN 4, B.Braun, Melsungen, Germany). After exposure of the dura, a subdural drainage (9-French drainage, Neuromedex, Hamburg, Germany) was inserted on one (left) side approximately 5–10 mm into the subdural space and guided through the skin. This drainage was later used for the subdural blood injection. This was followed by the bilateral transdural placement of a precalibrated multimodal intraparenchymal probe (Neurovent PTO, Raumedic AG, Helmbrechts, Germany; intracranial pressure (ICP) in mmHg, brain temperature (T_B_) in °C and brain oxygen partial pressure (P_bt_O_2_) in mmHg) about 1–1.5 cm subcortical. In addition, microdialysis catheters (CMA 600 Microdialysis Analyzer, CMA/Microdialysis AB, Harvard Bioscience Inc., Holliston, Massachusetts (MA), USA) were inserted bilaterally. After guiding the catheters and probes through the skin out, the dura and the drill holes were each sealed with Gelita-Spon (Gelita Medical AG, Eberbach, Germany) and bone wax (B.Braun, Germany). The wound was sutured.

**Figure 1:**
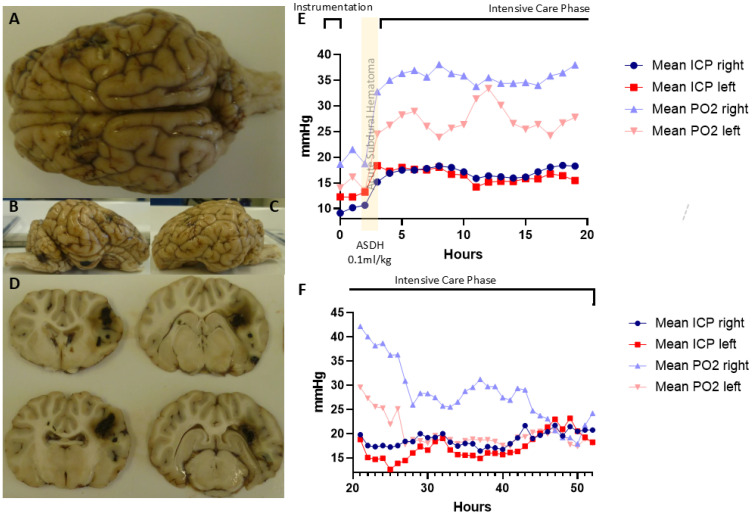
Macroscopic findings of a pig brain removed after acute subdural hematoma (ASDH) with the view (**A**) from above, (**B**) from the right, (**C**) from the left side and (**D**) in coronal sections from frontal to sub-occipital. In A–C fronto-parietal cortex after implantation of the neuro-monitoring probes and the right-sided ASDH. In (**D**), blood deposits on the right side of the cortex as evidence of intraparenchymal bleeding. (**E**,**F**): Exemplary courses of intracranial pressure (ICP) and partial oxygen pressure (PtO_2_, PO_2_) in mmHg over time for the right side (with ASDH) and the left side (control) of the animals analysed. There was an increase in ICP values and a decrease in the PtO_2_ values for the right ASDH hemisphere and subsequently for the control side after ASDH is applied (**E**). In the further course (**F**), ICP was significantly higher on the right than on the left side and the continuous drop of PtO_2_ values was more pronounced on the right than on the left.

A rest period of 60 min followed the neurosurgical instrumentation. In order to mirror the pre-clinical situation as much as possible, prior to the subdural blood application the FiO_2_ was reduced to 21%, the PEEP to 0 cm H_2_O, and the (I/E ratio) to 1:2. Autologous blood (0.1 mL/kg body weight) was then injected into the subdural space via the drainage tube over 15 min using an infusion pump (B.Braun, Germany). Systemic and multimodal neurosurgical monitoring (heart rate, blood pressure, arterial O_2_ saturation, cardiac output, ICP, T_B_ and P_bt_O_2_, as well as cerebral perfusion pressure (CPP = difference of mean arterial − intracranial pressure)) was performed continuously (Figure 1). Two hours after the induction of ASDH, TBI-targeted resuscitation [142] was initiated up to a total maximum experiment duration of 54 h after ASDH targeting baseline CPP levels. If necessary, continuous i.v. norepinephrine was administered to achieve this goal. Consciousness assessments were carried out every 24 h according to the Glasgow Coma Scale, specially modified for pigs [143]. For this purpose, the depth of anaesthesia was decreased until adequate spontaneous breathing resumed. At the end of the experiment, the anaesthesia was deepened and the animal euthanized with i.v. KCl. The microdialysis samples (microdialysate) were collected every 3 h and immediately evaluated (pyruvate, lactate, glucose and glutamate). Before. as well as at 2, 24, and 48 h after, induction of ASDH, anaesthesia serum samples were analysed for S100ß, Glial Fibrillary Acidic Protein (GFAP), neuron-specific enolase (NSE) and microtubule-associated protein 2 (MAP2) using specific kits (BlueGene BioTech, BioZol Diagnostics, Germany).

### 2.4. Analysis of Injury Patterns

**Figure 2:**
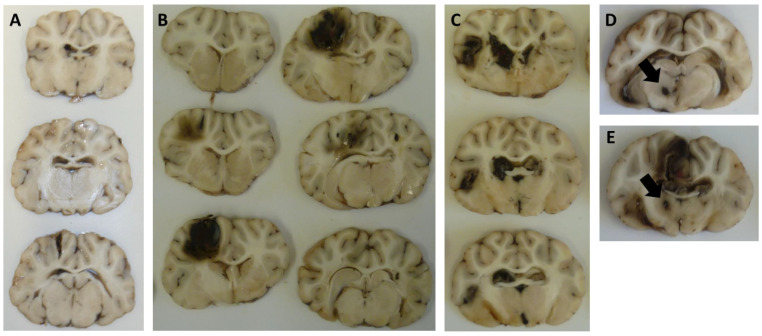
Injury pattern and distribution of haemorrhage after the experiment: (**A**) only extracerebral, (**B**) intraparenchymal–extraventricular, (**C**) intraventricular, (**D**) brain stem involvement (arrow) and (**E**) basal ganglia involvement (arrow).

Immediately postmortem, the skull and the dura were opened, and the brain was detached below the medulla oblongata and removed. The brain was cut in coronal standardized 5–6 mm slices from frontal to suboccipital and photo-documented. Brain samples were taken bilaterally in a standardized manner. Thereafter, the brain was fixed in 4% formalin for 6 days. The brain slices were then further sectioned (4 mm slices) and embedded in paraffin. The injury pattern was evaluated for the coronal slices according to (i) only extracerebral, (ii) intraparenchymal–extraventricular, and (iii) intraventricular location, as well as according to the involvement of (*A*) the brainstem and (*B*) the basal ganglia (Figure 2).

The occurrence of lesions of the basal ganglia or brainstem in close temporal association with TBI have an impact on consciousness and thus on clinical follow-up observations using the GCS, as well as on short-term and long-term outcome like survival [144,145,146,147]. For this reason, they played a role in the evaluation of this study. The intraparenchymal–extraventricular distribution could also include an extracerebral distribution, but not an intraventricular distribution. The same applies to the intraventricular distribution, which could also have had an extracerebral and intraparenchymal distribution. This classification should reflect the severity of the structural damage (see image-guided severity classifications) [23,24,25,26,27].

### 2.5. Primary, Secondary Endpoints and Outcome Evaluation

The primary endpoint of the present post-hoc analysis was reaching the end of the trial at the maximum of 54 h (52 h after ASDH). Whether the different patterns of injury and distribution of haemorrhage, as an expression of the different degrees of severity of experimental TBI, have an influence on survival in the experimental setting played a superordinate role. The duration of the experiment with living animals under conditions of an animal neuro-intensive care including multimodal neuromonitoring after ASDH as severe TBI represents the translational equivalent of human survival during post-traumatic therapy.

The secondary endpoint of this analysis was to identify the influence of the different injury patterns and blood distributions to clinically relevant treatment parameters, such as vital signs, temperature, anaesthesia parameters, blood gas analyses, neuromonitoring data and biomarkers in order to understand premature death of the animals. In humans, the pathophysiological and clinical processes described in the introduction, which are important for the outcome, only become clinically apparent after hours or days. It is therefore necessary to maximise the duration of the experiment in order to avoid possible confounding of the single-nodal targeting or focused (temporal) approach.

As described in detail in the introduction, the assessment of consciousness after TBI plays an essential role. Therefore, this should also be included in our experimental setup in the form of a modified GCS. However, current local animal welfare legislation does not allow the animal to be fully aroused from anaesthesia in this set-up to assess the maximum function of the brain. Therefore, this study was conducted according to a modified GCS with the following variables: assessment of consciousness (normal contact/response to environment, tendency to sleep, arousal response (e.g., eye opening) to visual, acoustic or physical stimuli, assessment of motor skill (normal, hemiparesis, extension, disturbed reflexes), and brainstem reflexes (normal, decreases pupil functions, bilateral enlarged pupils) (Table S1) [143]. In contrast to the modified GCS designed for dogs, the GCS modified by us for pigs is better suited to an examination situation in the prone position and with implanted probes and sensors [143]. Like the GCS for humans, a coma can be identified by a combination of values and not by a single constellation of values as in dogs. In line with the categorisation for dogs, this is adopted for pigs: Grade 3 = 3–8 points, Grade 2 = 9–14 points and Grade 1 = 15–18 points.

### 2.6. Statistical Evaluation

The statistical analyses were primarily used to differentiate between injury types and haemorrhage distributions with regard to the survival time of the animals in the experiment (translational approach for post-traumatic survival time in humans during intensive-care therapy) and secondary parameters such as consciousness or other factors such as cardiovascular or anaesthesia parameters. The Kolmogorov–Smirnov test was used to assess the normality of distribution of the investigated parameters. Due to the different occurrence of normally and non-normally distributed data and the relatively small groups, non-parametric analysis methods like the Kruskal–Wallis test or the Mann–-Whitney-U-test were used. Descriptive statistics are primarily used to describe the sample. Here the data set is described in its properties, such as the location parameters’ mean and median. Distribution parameters are described by variation range and quartiles. These results are presented in tables and graphs. For further evaluation, univariate evaluations (mean values, standard deviations), bivariate evaluations (diagrams, cross tables) and multidimensional evaluations (regression, testing of differences in variance) were carried out according to the cohort size. Kaplan–Meier analyses, including a log-rank test, were performed in the special observation of survival in the experimental set-up. In order to compare the time course of values (e.g., body temperature) from different injury or haemorrhage groups, mixed linear model analysis of variance was chosen, which takes into account the repeated measurement structure of the data (restricted maximum likelihood, REML). Residual analysis was included in order to check the presumptions of the model. The models (separately for basal ganglia injury, brainstem injury, haemorrhage type) included each the two main effects of time and group as well as their interaction. The independent variables for the regression models have been chosen based on the underlying research question, for which the primary focus was to evaluate a possible association between body temperature and both the time point of measurement and allocation to injury group. Thus, no variable selection process had to be defined or conducted. Specifically, this means that the variables used in this analysis are represented by the time of measurement, the group (injury versus no injury, or extracerebral versus intraparenchymal versus intraventricular) and the interaction effect of both. The regression equation was temperature~time+group+time*group. The outcome variable was always temperature. A logistic regression approach was used to investigate the influence of the injury patterns (brainstem injury and basal ganglia injury) and haemorrhage distribution on premature withdrawal of the animal from the experiment (binary endpoint). This means that the variables used in this analysis are represented by the occurrence of withdrawal before the planned experiment end (yes versus no; also, outcome variable), the group (injury versus no injury, or extracerebral versus intraparenchymal versus intraventricular) and the interaction effect. The regression equation was withdrawal~injuries+haemorrhage distributions+injuries*haemorrhage distributions. The variance infiltration factor (VIF) was used for the multicollinearity-check. The significance level was set to 0.05. All p-values are interpreted in an exploratory manner. The statistical analyses were carried out using SPSS 29 software (IBM, Armonk, NY, USA) and the R software for statistical computing (version 4.3.2, R Foundation for Statistical Computing, Vienna, Austria). GraphPad Prism 10 software (Boston, MA, USA) and Microsoft Office 365, PowerPoint software (Redmond, WA, USA) were used to create the graphs.

## 3. Results

### 3.1. Animal Characteristics and Injury Pattern

The characteristics of the animals, the distribution of the injury patterns and the amount of blood infused intracranially for ASDH are shown in Table 1. The majority of the test animals had intraventricular (*N* = 21, 47.7%) and intraparenchymal–extraventricular (*N* = 20, 45.5%) blood. A purely extracerebral blood distribution occurred in only a small number of animals (*N* = 3, 6.8%). There were *N* = 20 (45.5%) animals without and *N* = 24 (54.5%) animals with basal ganglia injuries. A mean of 11.8 (SD: 6.37) ml per animal was infused intracranially as ASDH. The amount of blood applied differed significantly between the groups according to the injury pattern. Animals with a purely extracerebral blood distribution had the largest amount of ASDH, whereas the mean ASDH volume was lower with intraparenchymal and intraventricular blood distribution, with a difference of 1.91 mL. There was no difference in the amount of blood infused subdurally between animals with brainstem injury (10.39 (SD:5.87) mL) and animals without brainstem injury (12.99 (SD:6.65) mL). The amount of subdural infused blood did not differ between animals without (12.83 (SD: 6.73) mL) and with (10.96 (SD: 6.07) mL) basal ganglia injuries. There is a difference in the amount of blood applied between the strains. (Table 1). The combined occurrence of the different parenchymal blood distributions for the occurrence of brainstem and basal ganglia lesions is shown in Table 1. Within the group with extracerebral blood distribution, there was no brainstem injury and no basal ganglia injury. Brainstem injuries and injuries to the basal ganglia were detected significantly more frequently with intraventricular blood distribution than with intraparenchymal blood distribution (Table 1). *N* = 18 (40.9%) animals had no brainstem and no basal ganglia injury. In *N* = 6 (13.6%) animals, only basal ganglia injuries occurred; in *N* = 2 (4.5%) animals only brainstem lesions occurred. Brainstem and basal ganglia injuries occurred in *N* = 18 (40.9%) experimental animals (Table 1).

Table 2 shows the incidence of injury patterns with regard to additional haemorrhagic shock as an additional injury. There was no significant difference in the distribution of injury patterns according to the intervention of haemorrhagic shock. There was no significant difference in the occurrence of brainstem or basal ganglia lesions between animals with and without haemorrhagic shock (Table 2).

### 3.2. Duration of Experiment

**Figure 3:**
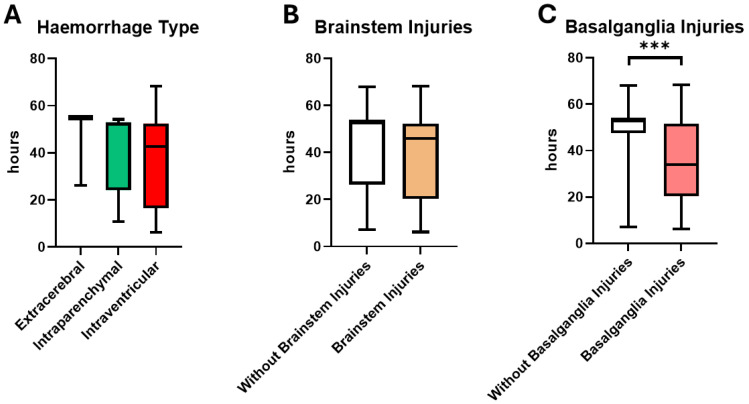
Median and interquartile representation of the duration of the experiment (survival of the animal) according to the division into (**A**) haemorrhage distributions, (**B**) occurrence of brainstem injuries and (**C**) occurrence of basal ganglia injuries. The significance of the difference between the injury patterns with and without basal ganglia injury in (**C**) is *p* = 0.0001 = *** (Mann–Whitney U test).

The course of the animals in the experiment as a surrogate of survival after TBI and as the primary endpoint of this post-hoc analysis is shown in Table 1. According to the pattern of haemorrhage, there was no significant difference in the duration of the experiment. However, animals with a purely extracerebral blood distribution tended to survive the experiment longer than the other groups. Animals with intraventricular blood distribution tended to die the earliest. Animals without brain stem injury and without basal ganglia injury stayed in the experiment longer than animals with such injuries (Figure 3).

**Figure 4:**
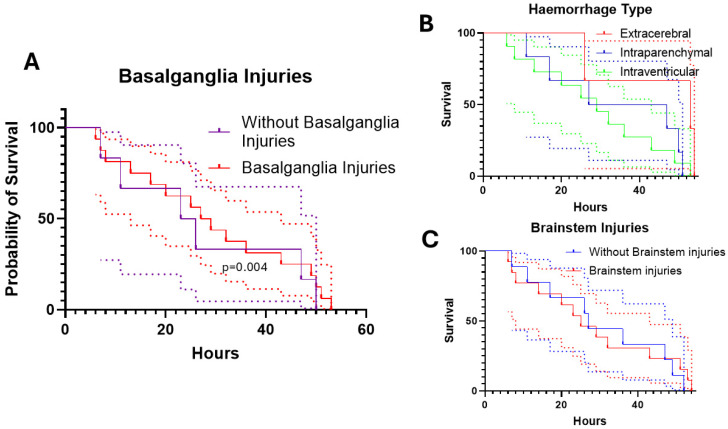
The exclusion of the animals from the experiment and the length of the experiment after application of the acute subdural hematoma. (**A**) There was a difference of animals with basal ganglia lesions and without basal ganglia injuries (*p* = 0.004, Log rank (Mantel–Cox)). (**B**) Animals with intraventricular blood distribution dropped out earlier than other animals, followed by animals with intraparenchymal blood distribution (*p* = 0.228, Log rank (Mantel–Cox). (**C**) Animals with brainstem lesions dropped out earlier than those without (*p* = 0.072, Log rank (Mantel–Cox). The dotted lines show the corresponding 95% confidence interval.

Figure 4 shows the survival of the animals in relation to the duration of the experiment and the time of death of the animals in relation to the pattern of injury. The single-injury pattern and the occurrence of brainstem lesions without any combination had no significant impact on the time in experiment. However, animals with basal ganglia lesions drop out significantly earlier (Figure 4). Comparing the injury patterns, the occurrence of brainstem damage and basal ganglia injuries, only the latter have an impact on early withdrawal from the experiment in the univariate (OR: 1.95 (CI:1.84–27.14), *p* = 0.004, R^2^ = 0.251) and multivariate (OR: 1.92 (CI:1.11–42.16), *p* = 0.038, R^2^ = 0.257) regression analysis.

### 3.3. Modified Glasgow Coma Scale

**Figure 5:**
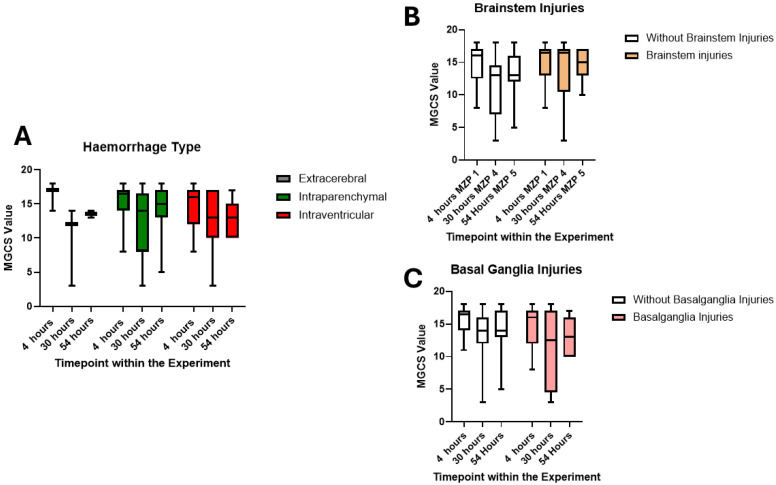
Total modified Glasgow Coma Scale scores (MGCS) over time (3 = minimum, 18 = maximum), showing scores at 4, 30 and 54 h of the current study (**A**–**C**). In general, the animals show a deterioration in scores after the application of trauma (acute subdural haematoma), in this case at hour 30, and then a recovery. Both non-extracerebral damage and damage to the brainstem and basal ganglia resulted in lower scores than without such damage. This suggests a clinical equivalent of brain damage.

Figure 5 shows the different courses of modified GCS (mGCS) values according to the different injuries and haemorrhage patterns. It can be seen that animals with intraparenchymal and intraventricular blood distribution show a different recovery on the mGCS compared to animals with extracerebral blood distribution. This can also be seen when subdividing animals with and without brainstem or basal ganglia injury. Due to the difficulties in obtaining data under anaesthesia, we did not calculate significant differences. Nevertheless, the clinical impact of the injury patterns can be seen in the course of this study, as is seen in humans.

### 3.4. Vital Parameters

**Figure 6:**
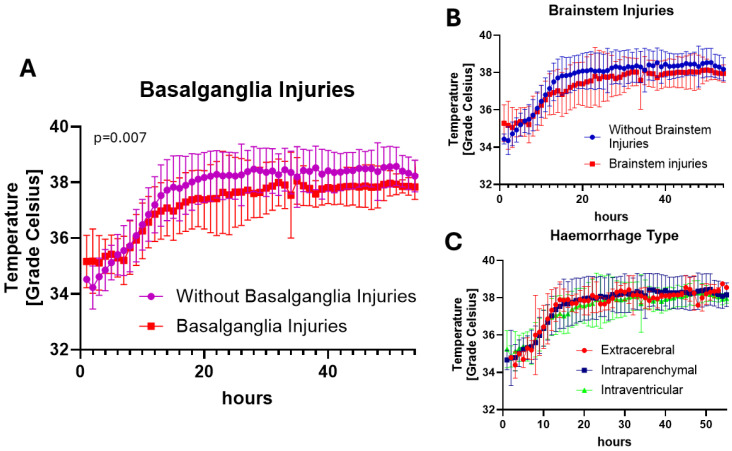
Body temperature of animals differentiated by (**A**) occurrence of basal ganglia injury, (**B**) occurrence of brainstem injuries and (**C**) different haemorrhage distribution. Results of the mixed-model approach (restricted maximum likelihood, REML): animals without basal ganglia injury showed higher body temperature than animals with basal ganglia injuries (*p* = 0.007) (**A**). The distribution for brainstem injuries and haemorrhage type revealed no significant results. The conditional R^2^ values are 0.489 for basal ganglia injury, 0.612 for brainstem injury and 0.609 for haemorrhage distributions.

The relationship between vital parameters and injury patterns is shown in Table 3. Differentiation into the injury patterns did not coincide with differences in the course of vital parameters (heart rate, body temperature and MAP at selected times points). However, the occurrence of basal ganglia injuries led to a high number of differences in vital signs such as heart rate, CVP and MAP. The occurrence of basal ganglia injury additionally shows a significant difference in body temperature (Table S2). Animals without basal ganglia injury, especially in the second half of the experiment, showed significantly higher body temperature than animals with basal ganglia (Figure 6).

### 3.5. Anaesthesia Parameters

Table 4 shows the differences between the various anaesthesia parameters in relation to the injury patterns. Significant differences in anaesthesia parameters appeared, especially for the parameters pPeak and respiratory minute volume (RMV). Animals with intraventricular haemorrhage had the highest and extraventricular (extracerebral and intraparenchymal) haemorrhage the lowest pPeak values (Table S3).

### 3.6. Blood Gas Analysis

Separation of the injury patterns and the occurrence of brainstem injuries or basal ganglia injury did not show any specific pattern for the blood gas analysis. (Tables S4 and S5).

### 3.7. Data of Neuro-Monitoring

**Figure 7:**
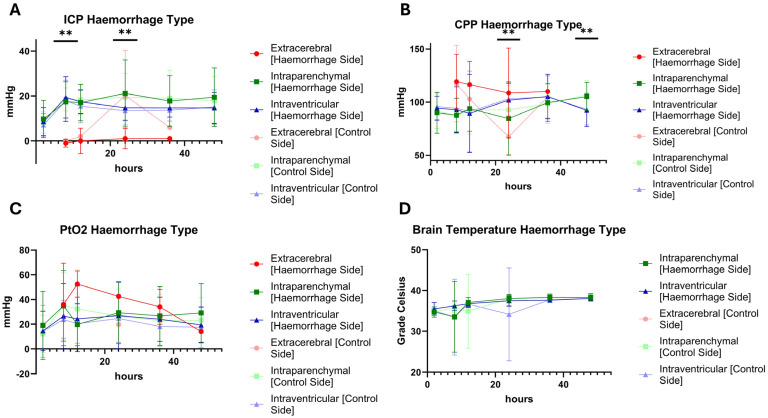
Time course of the neuromonitoring values over the experiment (0 to 54 h) separated by intraparenchymal and intraventricular haemorrhage. Extracerebral (*N* = 3) cases were excluded due to early dropout and the low number of values. (**A**) Intracranial pressure = ICP, (**B**) cerebral perfusion pressure = CPP, (**C**) partial tissue (brain) oxygen saturation = PtO2, (**D**) brain temperature in grade Celsius. ICP 8 h: haemorrhage side and control side and ICP 24 h: haemorrhage side, *p* ≤ 0.029 (**); Mann–Whitney-U Test (**A**). CPP 24 h: haemorrhage side, *p* = 0.05 (**) and CPP 48 h: control side, *p* = 0.046 (**); Mann–Whitney-U Test (**B**).

**Figure 8:**
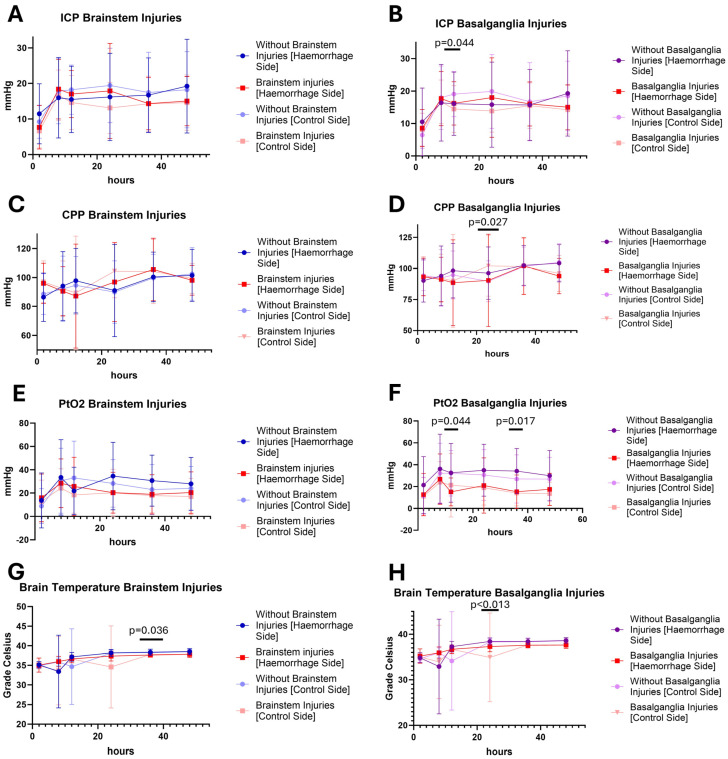
Time course of the neuromonitoring values over the experiment (0 to 54 h) separated by the occurrence of brainstem (**left**) and basal ganglia (**right**) injury. (**A**,**B**) Intracranial pressure = ICP, (**C**,**D**) cerebral perfusion pressure = CPP, (**E**,**F**) partial tissue (brain) oxygen saturation = PtO_2_, and (**G**,**H**) brain temperature in grade Celsius. Significant differences in animals with and without basal ganglia injury: ICP, *p* = 0.044 (12 h, control side) (**B**), CPP, *p* = 0.027 (24 h, control side) (**D**), PtO_2_, *p* = 0.044 (12 h, control side) and 0.017 (36 h, haemorrhage side) (**F**), temperature, *p* = 0.012 (24 h, haemorrhage side) and *p* = 0.002 (24 h, control side) (**H**). Significant differences in animals with and without brainstem injury: temperature, *p* = 0.036 (36 h, haemorrhage side) (**G**).

When considering the neuro-monitoring values, it is noticeable that animals with intraparenchymal bleeding often had higher ICP values than other injury patterns. In addition, these animals also had lower values for the cerebral perfusion pressure (CPP); although, these did do not always differ significantly (Figure 7). If the animals were differentiated according to the occurrence of brainstem injuries or injuries to the basal ganglia, there were only a few significant differences (Figure 8).

### 3.8. Biomarkers for Focal and Diffuse TBI

**Figure 9:**
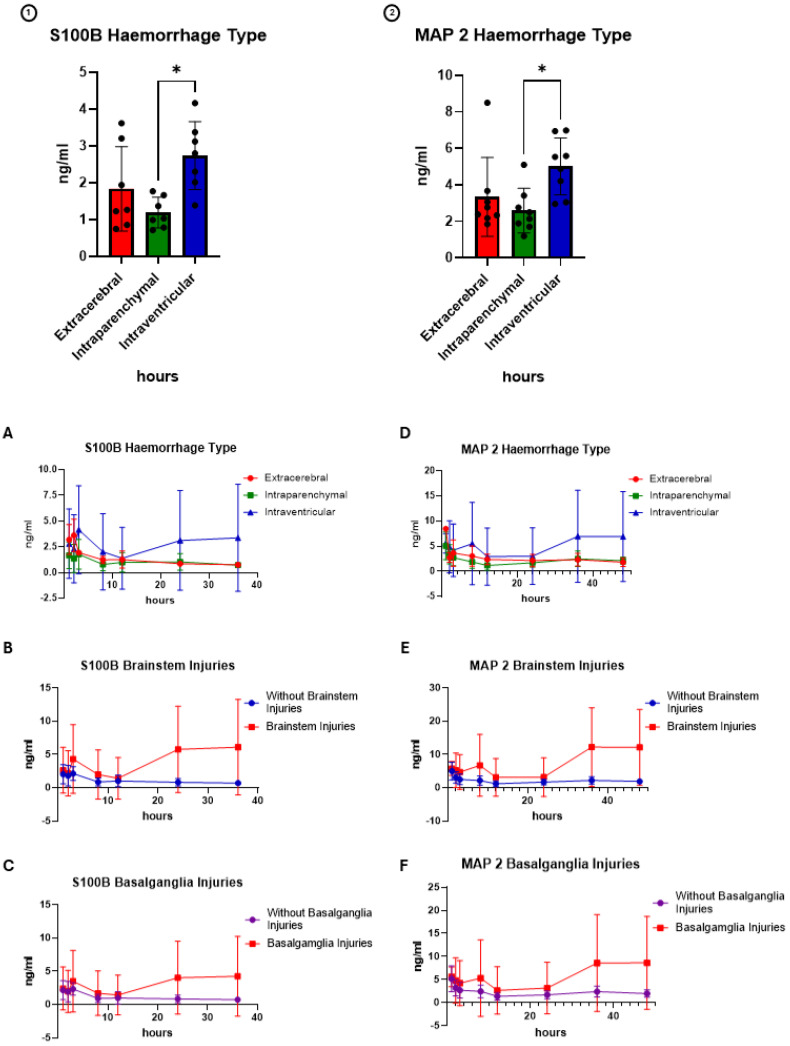
Time course of the biomarkers (1): S110ß and, (2): MAP2 separated based on the injury patterns. (**A**,**D**) Haemorrhage type, (**B**,**E**) occurrence of brainstem injuries and (**C**,**F**) occurrence of basalganglia injuries. The biomarkers S100ß (*p* = 0.0153 = *, Kruskal–Wallis-H) and MAP2 (*p* = 0.0126 = *, Kruskal–Wallis-H) showed significantly higher cumulative concentrations in animals with intraventricular injuries.

**Figure 10:**
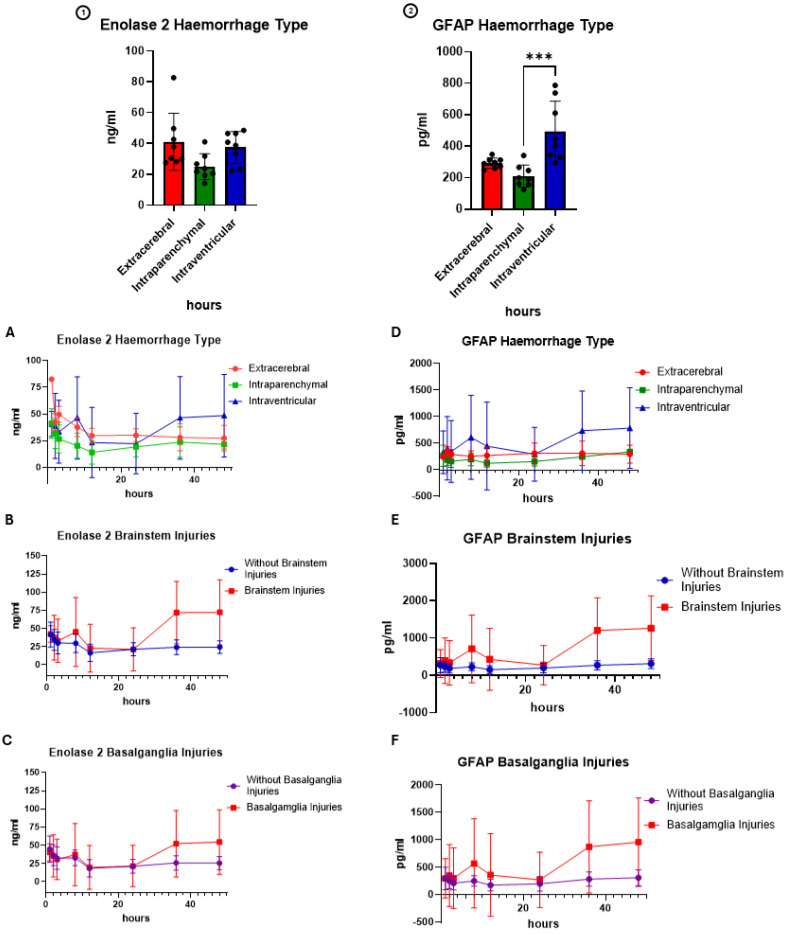
Time course of the biomarkers (1): NSE and (2): GFAP separated based on the injury patterns. (**A**,**D**) Haemorrhage type, (**B**,**E**) occurrence of brainstem injuries and (**C**,**F**) occurrence of basalganglia injuries. The biomarker GFAP (*p* = 0.0003 = ***, Kruskal–Wallis-H) showed significantly higher cumulative concentrations in animals with intraventricular injuries.

The results from the test for the biomarkers of glial cell injury (S100ß and GFAP) and neuronal cell injury (MAP2) are shown in Figure 9 and Figure 10. In contrast to the other injury patterns, there is a significant difference in the expression of biomarkers in animals with intracerebral haemorrhage patterns compared to other haemorrhage distributions or injury patterns.

In summary, this experimental design shows a majority of additional intraparenchymal and intraventricular injury patterns following application of ASDH as a TBI. More than half of the animals had additional injury to the basal ganglia. Animals with an intraventricular haemorrhage distribution tended to have the shortest survival time compared to animals with other haemorrhage distributions. The occurrence of basal ganglia injury was associated with more abnormalities in vital signs monitoring and the occurrence of intraventricular haemorrhage on anaesthesia parameters, as well as the frequency of increased ICP and decreased CPP values. Animals with an intracerebral haemorrhage component show significant differences from other haemorrhage and injury patterns in biomarkers of glial and neuronal cell injury.

## 4. Discussion

Traumatic brain injury (TBI) is the leading cause of adult mortality and morbidity in HICs [14,148]. Both focal and diffuse brain damage after TBI were subject to the same pathophysiological understanding of primary (early) and secondary (delayed) brain injury. Here, the primary brain injury is the direct consequence of the impact of force on the brain and its covering (head lacerations, fractures, contusions, bleeding). The secondary brain injury arises from processes of primary brain injury. However, these often only become apparent with a time lag to the primary brain injury and represent “cascades of biochemical, cellular and molecular events” like cerebral circulatory disorders, edema formation, destructive inflammation processes, disruption of the cellular homeostasis, ion channel disorders, increased intracranial pressure, ischemia and much more [149,150,151,152]. If the primary brain damage is survived, the phase of the secondary brain damage represents the more destructive phase. It is still poorly understood despite great scientific knowledge and is without any breakthrough in clinical innovations [153]. Since there is no direct clinical influence on the primary brain injury, the efforts are aimed towards the secondary brain injury to reduce morbidity and mortality after TBI. To improve clinical management, reduce mortality and improve outcomes, there is currently no translational experimental model available that reflects the individual circumstances of a TBI on an individual brain in such a way that insights could be derived from it, e.g., for drug therapy [22,99,154].

Our experimental setup represents a partially focal (ASDH) and partially diffuse (intra-, extraparenchymal, intraventricular, basal ganglia, brainstem) TBI. The haemorrhage distributions appear to reflect different degrees of severity of the TBI model according to survival times and modified GCS in the experiment. This assumption is supported by the fact that the presence of basal ganglia injury has an effect on other organ functions, such as heart rate, body temperature, CVP and MAP, and the presence of intraventricular haemorrhage has an effect on anaesthetic parameters, such as pPeak and RMV and especially ICP and CPP values. The latter are strongly associated with survival. They therefore have implications for clinical management strategies. Intraparenchymal injuries in particular show relevant biomarker changes. All this reflects the clinical situation in humans very well. The focus of our experimental setup is the pathophysiological cascade of the secondary brain injury through intracranial haemorrhage as in an acute subdural hematoma. We were able to show that this experimental setup simulates parts of the secondary brain injury by the fact that the ICP initially increased significantly, subsequently the serum levels of S100ß dropped significantly, and the formation of nitrotyrosine, albumin extravasation, NOX expression and the activation of microglia could be documented [133]. The purpose of this study was to describe the relevance of different injury patterns in this clinically relevant, long-term, resuscitated pig model of secondary brain injury after acute subdural hematoma.

### 4.1. Animal Models for Traumatic Brain Injuries

Animal models of brain injury mostly use young and healthy rodents, which have a lissencephalic brain and commonly lack standard intensive care [141,155,156,157]. Large animals with human-like brains may improve clinical translation due to similar neuroanatomical structure [158], and, in fact, swine are referred to be an ideal model species for brain injury studies due to their many similarities with humans that comprise a large brain mass, gyrencephalic cortex, high white matter volume and topography of basal cisterns, amongst other critical factors [101,141,157]. Moreover, ASDH represents a well-reproducible model of acute brain injury [159], which is based on the intracranial introduction of blood and the consecutive generation of ICP. This is able to trigger the pathophysiological process of secondary brain injury [133]. Thus, the core of this experimental setup also reflects one of the main pathophysiological processes following ASDH, since, in particular, the ICP increase is simulated [133,160]. Mouse and rat models of ASDH exist. However, these are difficult to perform due to the instrumentation (size of devices) and volume ratios and are difficult to transfer to humans in a translational sense [161,162]. Finally, using “human-sized” individual animals allows incorporating standard neurocritical care, which is well-established to significantly impact outcomes after moderate-to-severe acquired brain injury, and, thus, may reduce the translational gap for therapeutics and diagnostics, in particular for moderate-to-severe acquired brain injury [101,141,157]. Recently, we have developed a long-term, resuscitated model of ASDH-induced brain injury in human-sized pigs comprising bilateral, multimodal brain monitoring, integrating standard intensive care, intermittent neurological assessment by a modified GCS and measurement of blood biomarkers of brain injury, as well as post mortem morphological and immunohistochemical analyses [133]. Bilateral multimodal neurocritical care monitoring was performed to avoid sham experiments, i.e., the hemisphere without ASDH served as control for the hemisphere with ASDH, and thereby to comply with the 3R principle [133,163].

As with humans, there is not one, or always the same (e.g., force, force vector, cellular and molecular brain properties, immunostatus) TBI in animal experiments. TBI is a collection of different injuries with a common ending [164]. Therefore, results from different laboratories and different experiments cannot be exactly compared [156]. Our aim is to characterise the different injury patterns of our experiment and their influence on the experiment, and thus the secondary brain injury being studied. In the same experimental setup, the experiment yields an extracerebral, intraparenchymal and intraventricular haemorrhage pattern. There are numerous animal models of TBI [21,156,158,159,160,165,166,167,168,169,170,171,172,173,174,175,176,177]. These animal models can be differentiated according to the type of trauma into fluid percussion injury, controlled cortical impact injury, impact–acceleration models, non-impact rotational injury, penetrating brain injury, blast injury and repeated and combined models [156,167,175,178]. Experiments can also be divided into focal or diffuse injuries according to the general trauma pattern [172,175,178]. There are only few models that deal with the intracranial blood distribution after TBI and their consequences for result interpretation [160,179,180,181,182]. It is widespread to use mice and rats as test animals for TBI (simplicity of the surgical instrumentation, large number of test animals possible, group differences can be represented statistically, low purchase and maintenance costs). Large animals have the challenges of being ethically justifiable, complex surgical measures and behavioural tests, and having high personnel and financial costs [175]. Although there are numerous physiological, structural, and biological similarities between the human brain and those of non-human, higher developed mammals, there are limitations in interpreting the experimental results (trauma and histopathological response, gender differences) and translating them into clinical innovations [22,156].

Another challenge in animal models of TBI is predicting the outcome after trauma or the severity of the TBI. Although numerous scores exist to describe the severity of TBI in humans, the use of a score to assess consciousness and, thus, the severity of TBI in experimental animals is not widespread [16,172]. The degree of severity can be classified in rodents using the neurological severity score [183,184]. We decided to adapt the GCS for dogs to pigs. Using a GCS designed for dogs seemed plausible to us because conceptually it is very close to our test animal compared to mice. [143]. In the literature, the description of the mechanical injury pattern through a combination of histopathological, neurofunctional and behavioural investigations, as well as the investigation of molecular biomarkers, dominates [156]. Nevertheless, the description and evaluation of outcomes after TBI in humans also presents considerable challenges and is the subject of lively debate [8,185,186].

### 4.2. Injury Pattern of TBI in Humans

With a mortality rate of up to 67%, ASDH as a consequence of a high-speed or high-energy trauma is one of the most serious consequences of a TBI [187,188]. The treatment of ASDH is characterized by both medical (non-surgical) and surgical approaches, which are controversial because the prevailing guidelines are rarely supported by a higher level of evidence [69,86,87,127,164]. Traumatic intracerebral haemorrhages secondary to TBI account for 8 to 50% of cases in all, and especially in severe, TBI [78,189,190,191]. They represent a heterogeneous group of lesions. In the case of a small size after TBI, their relevance lies in the secondary increase in size during the secondary brain injury. This occurs in up to 75% of cases, with the result of space-occupying appearance, compression of eloquent brain areas and increased ICP [78,192]. This again shows that the duration of a translational experiment in TBI is very important in terms of different haemorrhage and injury patterns and the impact on secondary brain damage. A distinction is made between the early (primary brain injury) and delayed (secondary brain injury) occurrence of traumatic intracerebral haemorrhage [144,147,193]. Traumatic intraventricular bleeding accounts for up to 22% of all TBIs and is associated with a poor outcome (up to 70%) [194,195,196,197]. Regarding the course of our experiment, animals with intraventricular blood had the worst course in the experiment. It remains to be clarified whether the occurrence of intraparenchymal and intraventricular bleeding is related to the neurosurgical instrumentation and whether these are consequences of a peri-interventional coagulation disorder, or results of the acquisition of the ASDH [198,199]. The extent to which known differences in the arterial and venous blood supply between pigs and humans play a role in the development of the different injury patterns is unclear and has not yet been researched [200]. However, intraparenchymal and intraventricular haemorrhage occurred in our animals no matter the presence or absence of haemorrhagic shock due to haemorrhage. This indicates that the shock phase is not the leading factor in the appearance of these injury patterns.

### 4.3. Multimodal Neuromonitoring—Intracranial Pressure Monitoring (ICP)

According to current guidelines, there are recommendations for the introduction of ICP probes and external ventricular drainage (EVD) to monitor ICP in humans [69]. Accordingly, the placement of the ICP probe per se does not improve the outcome, but it does facilitate decision-making in the process of secondary brain injury. The recommendation to measure ICP (level IIB) and CPP (level IIB) using an ICP probe serves to reduce in-hospital and 2-week post-injury mortality [69]. New haemorrhages or enlargement of existing bleeding may occur during or after the placement of an EVD device, occurring either ipsilaterally on the side of the EVD or along the puncture canal. The frequency is described as 0.7% to 41% [201,202,203,204,205,206,207,208]. The majority of bleeding occurs as puncture tract haemorrhage and has a mean volume of 1.96 (SD:6.48) cm^3^ or 2 (SD:2.4) ml to 15 mL [205]. In the context of the placement of an intra-parenchymal (cranial) pressure probe, haemorrhage complications occur in 2.8% to 10% of cases [207,209,210,211,212]. In our experimental setup, intraparenchymal and intraventricular bleeding occurred in more than 40% of cases after ASDH application. The exact pathophysiology and therefore the origin of the haemorrhages, some of which were located in the brainstem and basal ganglia and remote from probes, is unclear. This requires examinations of coagulation and the temporal resolution of haemorrhage development within the experiment, e.g., by MRI [199].

### 4.4. Heterogeneity of TBIs and Individuality of Primary and Secondary Injury

The term TBI represents the accumulation of heterogeneous injuries of the brain and its coverings with individual courses [213,214]. This heterogeneity is also reflected in our approach to animal experiments. At this point, the topic and challenges of reproducibility of experimental results should not be discussed [215,216]. On the contrary, our results show that the three different injury patterns are reproducible. Our analyses of the experimental setup show that our animal model is a mixed TBI model, as it actually occurs in humans. Nevertheless, it is difficult to predict which injury pattern will occur when the experiment is carried out, since there are different individual conditions of primary (early) and secondary (delayed) brain damage depending on the animal. The robustness of the prediction of a specific injury pattern in our setup is low, but corresponds to the reality in humans [217]. In order to increase the reproducibility and robustness of the experiment, the neurosurgical instrumentations were carried out by three different surgeons in several time periods from the start. This should break up the one large experiment into several small experiments [218,219]. The instrumentation was carried out in different order of the surgeons.

In the future, imaging such as MRI should be incorporated into this experimental setup; for example, that would allow the use artificial intelligence to better identify injury patterns in similar anatomical conditions between humans and pigs and to better classify secondary brain damage [220]. Because of the anatomical and cellular similarity between humans and pigs, this animal is also suitable for answering the many questions about TBI treatment in children with open cranial sutures. In the future, the experimental setup should be adapted to a piglet to address, for example, the question of intracranial pressure in open cranial sutures and its effect on brain perfusion. This could provide valuable data on a minimum perfusion pressure [221].

### 4.5. Limitations of the TBI Model and the Analysis

Focal versus diffuse injury: due to the large selection of animal models and the dichotomization into either focal or diffuse trauma consequences, there does not seem to be an ideal animal model for TBI in humans, as it has been clinically shown that every human has an individual form of primary and thus secondary injury or has a combination of focal and diffuse damage [217]. Against this background, the reproducibility of animal experiments on TBI is a matter of intense debate [222,223]. Accordingly, our animal model cannot be divided into a purely focal or diffuse model. Here, too, it corresponds to the human situation. This TBI model is very personnel-intensive because it simulates the actual intensive care situation in humans, with the presence of an anaesthetist, a nurse and neurosurgeons for more than 54 h. It is also cost-intensive, from the equipment of an intensive care unit to the equipment with consumables directly on the animal and the cellular and biochemical analyses. We also find this in humans. Unlike humans, the animal cannot be fully aroused from anaesthesia. This limits the ability to assess the modified GCS as the best measure of brain function after TBI. Due to the lack of infrastructure, our experimental set-up did not allow diagnostic imaging of TBI using CT or MRI. This would be an important step towards better describing injury patterns between humans and animals, but also carries the risk of transport complications.

Criticism can be levelled at the statistical evaluation, as the numerous, detailed and usual statistical methods presented were not, for example, aligned again by Bayesian comparisons nor was an extensive examination of the statistical interactions between the factors identified carried out. However, after careful consideration, the authors are of the opinion that this analysis is exploratory in nature and that an overload of statistical details does not change or strengthen the translational message. The authors also point out that the relatively small number of animals used in the analysis means that there are various forms of variance or wide uncertainty bands.

## 5. Conclusions

We have been able to present an animal model with translational potential that simulates mixed focal and diffuse TBI, as seen in humans, and that can provide answers to currently pressing questions such as the controversies surrounding ICP management, conservative therapy for cerebral oedema and imaging of secondary brain damage. The experimental setup is anatomically, cellularly and molecularly close to humans, and as a large animal experiment, is able to conduct basic research, simulate clinical trials and, once the results have been applied, be adapted to clinical practice and community settings. In a cycle of basic research that is close to humans in terms of time (54 h) and setting (neuro-intensive care unit), findings from early clinical testing (Phase I, II trials) and the establishment of clinical practice (Phase III trials) can be fed back into the experiment to provide further insights for translation to the community level. In addition to the anatomical, cellular, molecular and intensive care proximity of this large animal experimental set-up to humans, the realistic duration of neuro-intensive care therapy after TBI (up to 54 h) and the influence of chronological and biological age, as well as comorbidities, gender, and when using very young experimental animals, TBI in children (e.g., characterisation of an increase in ICP and decrease in CPP after TBI with unclosed cranial sutures), can be investigated.

## Figures and Tables

**Table 1:** Animal characteristics, including strain (BMW = Bretoncelles–Meishan–Willebrand, FBM = familial hypercholesterolemia, Bretoncelles and Meishan) and details about age, sex, injury patterns, duration of experiment and reaching the intended experiment end (survival) as primary endpoint of the analysis. ASDH = acute subdural haematoma, ICU = intensive care unit, SD = standard deviation and TBI = traumatic brain injury. (^1^ = Chi^2^-Test; ^2^ = Kruskal–Wallis-H test, ^3^ = Mann–Whitney- U Test).

	N(%)	Extracerebral (*N* = 3, 6.8)	Intraparenchymal (*N* = 20, 45.5)	Intraventricular (*N* = 21, 47.7)	Total (*N* = 44)	*p*
BMW, N(%)	24 (55)	2 (4.5)	7 (15.9)	15 (34,1)	24 (100)	0.059 ^1^
FBM, N(%)	20 (45)	1 (2.3)	13 (29.5)	6 (13.6)	20 (100)	
Male, N(%)	25 (57)	1 (2.3)	15 (34.1)	9 (20.5)	25 (100)	0.081 ^1^
Female, N(%)	19 (43)	2 (4.5)	5 (11.4)	12 (27.3)	19 (100)	
Mean weight in kg (SD)		71.33 (6.66)	68.05 (11.75)	72.95 (9.62)	70.61 (10.58)	0.284 ^2^
Mean age in months (SD)		23.2 (21.41)	32.1 (15.05)	23.0 (13.71)	27.2 (15.16)	0.215 ^2^
Brainstem injury, N (%)		0 (0)	4 (9.1)	16 (36.4)	20 (45.5)	<0.001 ^1^
Basalganglia injury, N (%)		0 (0)	8 (18.2)	16 (36.4)	24 (54.5)	0.011 ^1^
Amount of subdural blood in ml (SD) [BMW 12.7 (6.29), FBM 10.7 (6.46); *p* = 0.042] ^3^		20 (0)	10.2 (6.00)	12.14 (6.36)	11.81 (6.37)	0.033 ^2^
Subdural blood with brainstem injury in ml (SD)		0 (0)	7.2 (3.13)	11.19 (6.18)	10.39 (5.87)	0.179 ^2^
Subdural blood with basalganglia injury in ml (SD)		0 (0)	10.29 (6.34)	11.30 (6.11)	10.39 (5.87)	0.319 ^2^
Duration of experiment in minutes (SD) and hours (SD) after ASDH		2724.33 (999.70) 45.41 (16.66)	2566.40 (915.49) 42.77 (15.26)	2213.67 (1233.09) 36.89 (20.55)	2408.82 (1077.56) 40.15 (17.96)	0.230 ^2^
Reaching the programmed scheduled hours (54 h) of TBI-targeted ICU treatment		2 (4.5)	10 (22.7)	6 (13.6)	18 (40.9)	0.251 ^1^

**Table 2:** Distribution of injury patterns according to isolated cranial trauma and the occurrence of haemorrhagic shock as a simulation of an additive injury in the post-hoc analysis. ASDH = acute subdural haematoma, BMW= Bretoncelles–Meishan–Willebrand, FBM= familial hypercholesterolemia, Bretoncelles and Meishan. (^1^ = Mann–Whitney- U Test; ^2^ = Chi2 test).

	Mean Weight kg (SD)	Mean ASDH Blood Volume mL (SD)	Distribution of Bleeding N (%)		Brainstem Injury N (%)	Basal Ganglia Injury N (%)
Group of intervention of haemorrhagic shocks, N = 34 (BMW N = 20, 59%; FBM N = 14, 41%)	72.2 (11.03)	9.4 (5.16)	extracerebral	1 (2.9)	18 (53)	21 (62)
			intraparenchymal	17 (50)		
			intraventricular	16 (47.1)		
Non-intervention group N = 10 (BMW N = 10, 100%)	65.2 (6.83)	20 (0)	extracerebral	2 (20)	2 (20)	3 (30)
			intraparenchymal	3 (30)		
			intraventricular	5 (50)		
*p*	0.024 ^1^	<0.001 ^1^	0.136 ^2^		0.066 ^2^	0.076 ^2^

**Table 3:** Injury patterns and vital parameters over the course of the experiment. Mean arterial pressure = MAP.

		Number of Hours in the Experiment							
Parameter	Group	1	2	3	8	12	24	36	48
Vital parameters									
Heart Rate [beats × min^−1^] (SD)	Extracerebral	67.(17)	66.(2)	69 (12)	79(23)	95 (37)	83 (46)	66 (10)	70(20)
	Intraparenchymal	76 (22)	72 (17)	69 (20)	91 (29)	85 (31)	94 (38)	93(37)	85 (30)
	Intraventricular	66(26)	73 (23)	66 (18)	91 (37)	84 (28)	81 (22)	77 (10)	78 (12)
Body temperature [Grade Celsius] (SD)	Extracerebral	35.2 (0.1)	35.0 (0.5)	34.6 (0.5)	35.7 (1.4)	37.0 (0.9)	37.2 (0.7)	37.8 (0.3)	38.2 (0.6)
	Intraparenchymal	34.8 (0.5)	34.8 (1.3)	35.0 (1.1)	35.5 (0.9)	37.0 (1.0)	38.0 (1.2)	38.3 (0.9)	38.2 (0.9)
	Intraventricular	34.9 (0.6)	34.9 (0.6)	35.0 (0.6)	35.9 (0.9)	37.1 (1.0)	38.4 (1.2)	38.3 (0.6)	38. (0.5)
MAP [mmHg] (SD)	Extracerebral	99 (21)	96 (14)	104 (4)	118 (7)	123 (11)	114 (15)	117(12)	113 (11)
	Intraparenchymal	107(18)	86 (33)	98 (25)	101 (23)	111 (18)	110 (23)	116 (16)	119 (16)
	Intraventricular	101(16)	100 (18)	103 (15)	99 (40)	112 (22)	121 (16)	123 (16)	71 (50)

**Table 4:** Injury patterns and anaesthesia values over the course of the experiment. Peak airway pressure = pPeak, positive end-expiratory pressure = PEEP.

			Hours							
Parameter (SD)	Group	1	2	3	8	12	24	36	48	*p*
Anaesthesia										
Respiratory rate [× min^−1^]	Extracerebral	8 (0)	8 (0)	8 (0)	8 (0)	10 (2)	10 (3)	8 (0)	8 (0)	
	Intraparenchymal	9 (1)	8 (1)	8 (1)	11 (3)	12 (3)	11 (2)	10 (2)	10 (2)	
	Intraventricular	9 (1)	9 (1)	8 (1)	11 (3)	10 (2)	11 (3)	10 (2)	10 (2)	
Respiratory minute volume [L × min^−1^]	Extracerebral	4.6 (0.6)	4.5 (0.6)	4.5 (0.6)	4.6 (0.6)	5.7 (2.0)	6.1 (2.8)	4.4 (0.5) *	4.2 (0)	0.019 36 h 0.043 48 h
	Intraparenchymal	4.8 (0.8)	4.7 (0.7)	4.6 (0.8)	5.9 (1.4)	6.6 (1.9)	6.0 (1.3)	5.7 (1.3)	5.5 (1.3) *	
	Intraventricular	5.2 (0.9)	5.1 (0.8)	5.0 (0.7)	6.5 (1.8)	6.3 (1.2)	6.4 (0.9)	6.4 (1.0) *	6.0 (1.0) *	
Ppeak [cmH20]	Extracerebral	15 (1)	15 (1)	16 (3)	17 (7)	22 (2)	21 (2) *	20 (1) *	19 (0)	0.035 24 h 0.011 36 h
	Intraparenchymal	17 (2)	17 (4)	18 (5)	20 (5)	23 (2)	23 (2)	22 (2)	23 (2)	
	Intraventricular	17 (2)	17 (3)	17 (4)	21 (6)	23 (3)	24 (2) *	24 (2) *	24 (5)	
PEEP [mmHg]	Extracerebral	4 (1)	4 (1)	4 (1)	5 (5)	8 (3) *	9 (1)	10 (0)	10 (0)	0.043
	Intraparenchymal	6 (2)	6.06 (3)	5.24 (4)	7.50 (5)	9.90 (1) *	10 (1)	10 (0)	10 (0)	
	Intraventricular	5 (2)	5.0 (3)	4.0 (4)	7.53 (4)	10.0 (0) *	10.0 (0)	10 (0)	10 (0)	

* indicates which values significantly differ from each other.

## Data Availability

The datasets generated and/or analysed during the current study are not publicly available due local institutional property rights but are available from the corresponding author on reasonable request.

## Supplementary Materials

The following supporting information can be downloaded at: https://www.mdpi.com/article/10.3390/cells14010017/s1, Table S1: Presentation of the assessment of the Glasgow Coma Scale in humans according to Jennett and Teasdale [16], in dogs according to Platt et al. [143] and in pigs by our working group. In humans, a veritable GCS can only be determined without sedation and with physiological circulatory values. In pigs, an adaptation of the dog scoring is carried out, as the experimental setup does not allow complete awakening and performance of the oculocephalic reflex test. In contrast to humans, in which wakefulness reactions are tested, in animals it is primarily neurological systems that are examined. In dogs, the definition of coma is done by one value of the consciousness variable. As in humans, adaptation for pigs defines coma by a combination of variables and a value <9. Table S2: Significant results in vital parameters differentiating between animals with and without brainstem injuries and basal ganglia injuries. Table S3: Significant results in anaesthesia parameters differentiating between different injury patterns and with and without brainstem injuries and basal ganglia injuries. Peak airway pressure = pPeak, Positive End-Expiratory Pressure = PEEP, Fraction of inspiratory Oxygen = FiO2, Endtidal Oxygenpressure = EtO2, Partial Oxygenpressure = SpO2, Respiratory minute volume = RMV, Respiratory frequency = RF. Table S4: Significant results in blood gas analysis parameters differentiating between different injury patterns and with and without brainstem injuries and basal ganglia injuries. Pondus Hydrogenii = pH, concentration of Lactate = cLacta, concentration of Natrium, Partial pressure of CO2 = pCO2, Partial pressure of Oxygen = pO2, Oxygen pressure of 50% saturated Oxygen = p50e, Fraction of Met-Haemoglobin = FMetHB, concentration of Kalium, concentration of total Haemoglobin, concentration of Calcium = cCa, Fraction of deoxyhaemoglobin = FHHb, concentration of total oxygen ctO2c, concentration of Glucose = cGluv, Haematocrit = Hctc, Standard Base-Excess cBase (Ecf), Standard Bicarbonate = cHCO3. Table S5: Blood gas analysis results over the course according to selected time points. Pondus Hydrogenii = pH, concentration of Lactate = cLacta, concentration of Natrium, Partial pressure of CO2 = pCO2, Partial pressure of Oxygen = pO2, Oxygen pressure of 50% saturated Oxygen = p50e, Fraction of Met-Haemoglobin = FMetHB, concentration of Kalium, concentration of total Haemoglobin, concentration of Calcium = cCa, Fraction of deoxyhaemoglobin = FHHb, concentration of total oxygen ctO2c, concentration of Glucose = cGluv, Haematocrit = Hctc, Standard Base-Excess cBase (Ecf), Standard Bicarbonate = cHCO3.
