# Supplementary material for: Unravelling Secondary Brain Injury: Insights from a Human-Sized Porcine Model of Acute Subdural Haematoma
Source: Cells. 2024 Dec 27;14(1):17. doi: 10.3390/cells14010017 (PMC11720468; doi:10.3390/cells14010017)
Supplement: Supplementary file 1 [file cells-14-00017-s001.zip › Supplement Table S1 Rev 2.pdf]

Supplementary Material 1: Presentation of the assessment of the Glasgow Coma Scale in humans according to Jennett and Teasdale (16), in dogs according to Platt et al. (143) and in pigs by our working group. In humans, a veritable GCS can only be determined without sedation and with physiological circulatory values. In pigs, an adaptation of the dog scoring is carried out, as the experimental setup does not allow complete awakening and performance of the oculocephalic reflex test. In contrast to humans, in which wakefulness reactions are tested, in animals it is primarily neurological systems that are examined. In dogs, the definition of coma is done by one value of the consciousness variable. As in humans, adaptation for pigs defines coma by a combination of variables and a value <9.

	**Humans [16]**			**Dogs [143]**			**Pigs**		
**Points**	**Best eye**	**Best verbal**	**Best motor**	**Consciousness**	**Brainstem**	**Motor**	**Consciousness**	**Brainstem**	**Motor**
1	None	None	None	Comatose, unresponsive to repeated noxious stimuli	Bilateral, unresponsive mydriasis with reduced to absent oculocephalic reflexes	Recumbent, hypotonia of muscle, depressed or absent spinal reflexes	No reaction	Bilateral fixed mydriasis, no corneal reflexes	Hypotonia of muscle, depressed or absent spinal reflexes (side position)
2	Opening to pain	(incompre-hensible) Sounds	Pathological extending	Semicoma, responsive only to repeated noxious stimuli	Unilateral unresponsive mydriasis with reduced to absent oculocephalic reflexes	Recumbent, constant extensor rigidity with opisthotonus	responsive only to repeated physical stimuli	Unilateral fixed mydriasis	Persistent extensor rigidity with opisthotonus (side position)
3	Opening to speech	(inappropriate) Words	Abnormal flexing	Semicoma, responsive to auditory stimuli	Pinpoint pupils, reduced to absent oculocephalic reflexes	Recumbent constant extensor rigidity	Responsive to auditory stimuli	Pinpoint pupils	Persistent extensor rigidity (side position)
4	Spontaneous	Confused, disorientated	Mass movement	Semicoma, responsive to visual stimuli	Bilateral unresponsive miosis with normal to reduced oculocephalic reflexes	Recumbent, intermittent extensor rigidity	Responsive to visual stimuli	Bilateral unresponsive miosis	intermittent extensor rigidity (side position)
5	-	Orientated	Localising	Depression or delirium, capable of responding but response may be inappropriate	Slow pupillary reflexes and normal to reduced oculocephalic reflexes	Hemiparesis, tetraparesis or decerebrate activity	Somnolent, drowsy	Reduced pupillary reflexes	Hemiparesis or Tetraparesis
6	-	-	Obeys commands	Occasional periods of alertness and responsive to environment	Normal pupillary and oculocephalic reflexes	Normal gait, spinal reflexes	Normal, adequate contact to the environment	Normal pupillary reflexes	Normal spinal reflexes
