# Supplementary material for: Unravelling Secondary Brain Injury: Insights from a Human-Sized Porcine Model of Acute Subdural Haematoma
Source: Cells. 2024 Dec 27;14(1):17. doi: 10.3390/cells14010017 (PMC11720468; doi:10.3390/cells14010017)
Supplement: Supplementary file 1 [file cells-14-00017-s001.zip › Supplement Table S2 Rev 2.pdf]

Brainstem injury

Hours	Parameter	Brainstem injury	Mean (SD)	p
4	MAP	no	117.19 (18.29)	0.006
		yes	108.43 (13.63)	
49	MAP	no	119.25 (19.65)	0.038
		yes	113.33 (13.57)	
51	MAP	no	125.45 (15.89)	0.038
		yes	111.50 (12.23)	

Basal ganglia injury

Hours	Parameter	Basal ganglia injury	Mean (SD)	p	Hours	Parameter	Basal ganglia injury	Mean (SD)	p
15	Temp.	no	37.86 (1.09)	0.034	4	MAP	no	120.05 (17.76)	0.039
		yes	36.99 (1.08)				yes	108.76 (15.05)	
18	Temp.	no	38.06 (0.98)	0.047	5	MAP	no	112.74 (25.74)	0.002
		yes	37.37 (1.06)				yes	85.45 (28.69)	
19	Temp.	no	38.14 (0.94)	0.03					
		yes	37.44 (0.98)		7	CVP	no	2.61 (4.49)	0.005
20	Temp.	no	38.18 (0.97)	0.022			yes	6.65 (4.27)	
		yes	37.42 (0.99)		9	CVP	no	6.74 (3.96)	0.034
21	Temp.	no	38.23 (0.98)	0.028			yes	9.17 (3.69)	
		yes	37.46 (1.16)		15	CVP	no	8.12 (3.72)	0.029
22	Temp.	no	38.27 (0.95)	0.041			yes	10.32 (2.34)	
		yes	37.49 (1.26)		18	CVP	no	8.0 (4.62)	0.049
28	Temp.	no	38.46 (0.89)	0.007			yes	10.58 (2.76)	
		yes	37.68 (0.83)		21	CVP	no	8.22 (4.66)	0.023
29	Temp.	no	38.40 (0.88)	0.027			yes	11.44 (3.11)	
		yes	37.72 (0.86)		22	CVP	no	8.33 (4.56)	0.05
34	Temp.	no	38.34 (0.78)	0.043			yes	10.71 (2.29)	
		yes	37.56 (1.54)		23	CVP	no	8.33 (4.13)	0.017
37	Temp.	no	38.38 (0.78)	0.034			yes	10.94 (2.19)	
		yes	37.81 (0.61)						
38	Temp.	no	38.5 (0.81)	0.008	1	Heart rate	no	63.61 (29.73)	0.01
		yes	37.63 (0.47)				yes	77.74 (12.92)	
40	Temp.	no	38.39 (0.76)	0.04	11	Heart rate	no	92.53 (28.79)	0.037
		yes	37.85 (0.56)				yes	76.42 (27.92)	
42	Temp.	no	38.35 (0.69)	0.048	23	Heart rate	no	106.06 (34.72)	0.05
		yes	37.86 (0.60)				yes	83.63 (34.49)	
43	Temp.	no	38.39 (0.67)	0.055	34	Heart rate	no	90.63 (33.89)	0.041
		yes	37.88 (0.69)				yes	69.69 (34.49)	
44	Temp.	no	38.41 (0.67)	0.048	35	Heart rate	no	99.0 (36.07)	0.035
		yes	37.88 (0.67)				yes	75.46 (22.30)	
46	Temp.	no	38.47 (0.63)	0.045	44	Heart rate	no	91.88 (28.94)	0.029
		yes	37.89 (0.77)				yes	71.18 (15.04)	
47	Temp.	no	38.52 (0.68)	0.02	55	Heart rate	no	110.21 (41.30)	0.051
		yes	37.87 (0.77)				yes	75.50 (16.84)	
48	Temp.	no	38.59 (0.80)	0.029	56	Heart rate	no	115.07 (35.25)	0.052
		yes	37.87 (0.73)				yes	78.67 (19.92)	
49	Temp.	no	38.56 (0.76)	0.042					
		yes	37.96 (0.56)						
50	Temp.	no	38.59 (0.74)	0.018					
		yes	37.99 (0.43)						
51	Temp.	no	38.59 (0.71)	0.022					
		yes	37.97 (0.37)						
52	Temp.	no	38.4 (0.59)	0.024					
		yes	37.92 (0.29)						
