# Supplementary material for: Unravelling Secondary Brain Injury: Insights from a Human-Sized Porcine Model of Acute Subdural Haematoma
Source: Cells. 2024 Dec 27;14(1):17. doi: 10.3390/cells14010017 (PMC11720468; doi:10.3390/cells14010017)
Supplement: Supplementary file 1 [file cells-14-00017-s001.zip › Supplement Table S3 Rev 2.pdf]

**Injury pattern**									
Hours	Parameter	Injury pattern	Mean (SD)	p	Hours	Parameter	Injury pattern	Mean (SD)	P
15	pPeak	EC	22.0 (2.65)		4	FiO2	EC	0.32 (0.03)*	
		IP	22.55 (2.37)*	0.023			IP	0.23 (0.04)*	0.012
		IV	24.0 (1.66)*				IV	0.24 (0.05)*	
16	pPeak	EC	22.5 (2.12)						
		IP	21.60 (5.43)*	0.014	4	PEEP	EC	4.33 (1.16)*	
		IV	24.25 (1.57)*				IP	1.05 (2.52)*	0.022
17	pPeak	EC	20.33 (0.58)*				IV	3.25 (4.24)*	
		IP	22.58 (2.93)	0.004	10	PEEP	EC	8.33 (2.89)*	
		IV	24.25 (1.88)*				IP	10 (0)*	0.038
18	pPeak	EC	19.67 (1.53)*				IV	9.76 (1.09)*	
		IP	21.84 (3.92)	0.005	11	PEEP	EC	8.33 (2.89)*	
		IV	24.06 (1.92)*				IP	9.90 (0.45)*	0.035
19	pPeak	EC	19.67 (1.53)*				IV	10.0 (0)*	
		IP	22.47 (2.29)	0.011	12	PEEP	EC	8.33 (2.89)*	
		IV	23.69 (1.89)*				IP	9.90 (0.45)*	0.043
20	pPeak	EC	20.0 (1.0)*				IV	10.0 (0)*	
		IP	23.0 (3.06)	0.008	14	PEEP	EC	8.33 (2.89)*	
		IV	24.33 (1.80)*				IP	9.90 (0.45)*	0.047
21	pPeak	EC	19.67 (1.16)*				IV	10.0 (0)*	
		IP	22.53 (2.32)	0.004	15	PEEP	EC	8.33 (2.89)*	
		IV	24.06 (1.84)*				IP	9.90 (0.45)*	0.047
22	pPeak	EC	20.0 (1.0)*				IV	10.0 (0)*	
		IP	22.50 (2.66)	0.008	16	PEEP	EC	7.50 (3.54)*	
		IV	23.75 (1.84)*				IP	10.15 (1.23)*	0.042
23	pPeak	EC	21.0 (2.0)*				IV	10 (0) *	
		IP	22.44 (2.01)	0.024	32	PEEP	EC	9.33 (1.16)*	
		IV	24.00 (1.97)*				IP	10.0 (0)*	0.011
24	pPeak	EC	21.00 (2.0)*				IV	10.0 (0)*	
		IP	22.56 (2.36)	0.035					
		IV	24.00 (2.07)*		33	RMV	EC	4.30 (0.42)*	
25	pPeak	EC	20.67 (1.53)*				IP	5.81 (1.61)	0.031
		IP	22.61 (2.33)	0.017			IV	6.49 (1.22)*	
		IV	24.27 (2.34)*		34	RMV	EC	4.35 (0.50)*	
27	pPeak	EC	20.67 (1.53)*				IP	5.81 (1.73)	0.034
		IP	22.78 (2.82)	0.008			IV	6.35 (1.08)*	
		IV	24.67 (2.47)*		35	RMV	EC	4.35 (0.50)	
28	pPeak	EC	20.67 (1.53)*				IP	5.45 (1.09)	0.007
		IP	23.00 (3.05)	0.028			IV	6.41 (0.96)*	
		IV	24.47 (2.62)*		36	RMV	EC	4.35 (0.50)*	
29	pPeak	EC	20.67 (1.53)*				IP	5.65 (1.33)	0.019
		IP	22.82 (3.86)	0.015			IV	6.36 (1.02)*	
		IV	24.40 (2.35)*		37	RMV	EC	4.40 (0.57)*	
31	pPeak	EC	20.67 (1.53)*				IP	5.63 (1.30)	0.027
		IP	21.64 (2.06)	0.007			IV	6.28 (1.06)*	
		IV	24.60 (2.80)*		39	RMV	EC	4.40 (0.57)*	
32	pPeak	EC	20.33 (1.53)				IP	5.59 (1.28)	0.022
		IP	21.50 (2.93)*	0.014			iv	6.32 (0.87)*	
		IV	24.23 (2.65)*		40	RMV	EC	4.35 (0.50)*	
33	pPeak	EC	19.50 (0.71)				IP	5.51 (1.27)	0.023
		IP	21.93 (1.54)*	0.004			IV	6.06 (0.77)*	
		IV	24.38 (2.66)*		41	RMV	EC	4.35 (0.50)*	
34	pPeak	EC	19.50 (0.71)				IP	5.54 (1.26)	0.009
		IP	22.14 (1.61)*	0.007			IV	6.16 (0.74)*	
		IV	24.31 (2.63)*		42	RMV	EC	4.35 (0.50)*	
35	pPeak	EC	19.50 (0.71)*				IP	5.54 (1.27)	0.015
		IP	22.23 (1.79)	0.011			IV	6.03 (0.75)*	
		IV	24.15 (2.30)*		43	RMV	EC	4.90 (0.29)*	
36	pPeak	EC	19.50 (0.71)*	0.011			IP	5.61 (1.25)	0.026
		IP	22.07 (1.64)*				IV	6.07 (5.74)*	

		IV	23.64 (1.69)*		44	RMV	EC	4.80 (0.42)*	
37	pPeak	EC	19.50 (0.71)*				IP	5.51 (1.17)	0.031
		IP	21.50 (1.95)*	0.003			IV	5.95 (0.80)*	
		IV	23.75 (1.66)*		45	RMV	EC	4.80 (0.42)*	
38	pPeak	EC	19.50 (0.71)*				IP	5.49 (1.30)	0.025
		IP	22.43 (1.65)*	0.014			IV	6.02 (0.78)*	
		IV	23.73 (1.56)*		46	RMV	EC	4.56 (0.78)*	
39	pPeak	EC	19.50 (0.71)*				IP	5.46 (1.30)	0.02
		IP	22.57 (1.83)*	0.026			IV	6.21 (0.97)*	
		IV	23.55 (1.70)*		47	RMV	EC	4.60 (0.57)*	
40	pPeak	EC	19.50 (0.70)*				IP	5.54 (1.24)	0.018
		IP	22.64 (1.99)*	0.03			IV	6.16 (0.86)*	
		IV	23.73 (1.74)*		48	RMV	EC	4.20 (0)*	
41	pPeak	EC	19.50 (0.71)*				IP	5.48 (1.27)	0.043
		IP	22.71 (2.02)*	0.044			IV	5.99 (0.95)*	
		IV	23.60 (1.78)*		49	RMV	EC	4.85 (1.06)*	
42	pPeak	EC	19.50 (0.71)*				IP	5.50 (1.23)	0.05
		IP	22.71 (2.13)*	0.018			IV	6.05 (0.77)*	
		IV	23.91 (1.58)*		50	RMV	EC	4.85 (1.06)*	
46	pPeak	EC	20.0 (1.41)*				IP	5.56 (1.23)	0.026
		IP	22.64 (2.44)	0.046			IV	6.25 (0.85)*	
		IV	24.00 (2.05)*		51	RMV	EC	4.85 (1.06)*	
							IP	5.47 (0.99)*	0.017
2	EtCO2	EC					IV	6.37 (0.83)*	
		IP	39.56 (2.92)	0.027	52	RMV	EC	4.85 (1.06)	
		IV	35.08 (5.92)				IP	5.46 (1.05)*	0.027
3	EtCO2	EC					IV	6.30 (0.81)*	
		IP	40.88 (3.05)	0.018	53	RMV	EC	4.85 (1.06)	
		IV	34.23 (6.34)				IP	5.46 (1.05)*	0.038
8	EtCO2	EC	47.00 (0)				IV	6.30 (0.81)*	
		IP	38.00 (5.97)*	0.03					
		IV	32.21 (7.28)*						
28	EtCO2	EC							
		IP	44.17 (4.02)	0.035					
		IV	38.11 (6.39)						
29	EtCO2	EC							
		IP	44.17 (3.40)	0.05					
		IV	38.56 (6.48)						
32	EtCO2	EC							
		IP	44.18 (3.09)	0.012					
		IV	40.44 (3.71)						
37	EtCO2	EC							
		IP	46.00 (5.85)	0.021					
		IV	40.25 (3.54)						
38	EtCO2	EC							
		IP	43.91 (5.85)	0.018					
		IV	40.25 (3.54)						

Brainstem injury									
Hours	Parameter	Brainstem injury	Mean (SD)	p	Hours	Parameter	Brainstem injury	Mean (SD)	P
5	pPeak	no	13.26 (2.56)	0.049	1	RMV	no	4.64 (0.76)	0.007
		yes	15.11 (2.85)				yes	5.38 (0.76)	
6	pPeak	no	13.83 (2.66)	0.04	2	RMV	no	4.67 (0.69)	0.049
		yes	15.8 (3.97)				yes	7.90 (12.15)	
7	pPeak	no	16.0 (5.04)	0.011	8	RMV	no	5.49 (1.18)	0.021
		yes	19.35 (5.20)				yes	6.75 (1.81)	
8	pPeak	no	18.79 (4.98)	0.024	40	RMV	no	5.44 (1.33)	0.026
		yes	22.10 (4.87)				yes	5.96 (0.66)	
10	pPeak	no	22.79 (3.06)	0.013	41	RMV	no	5.51 (1.32)	0.045
		yes	23.80 (1.70)				yes	5.98 (0.69)	
11	pPeak	no	22.83 (2.39)	0.017	42	RMV	no	5.49 (1.34)	0.036
		yes	24.05 (1.64)				yes	5.89 (0.64)	
12	pPeak	no	22.30 (1.87)	0.007	43	RMV	no	5.62 (1.27)	0.044
		yes	23.56 (2.68)				yes	5.92 (0.64)	
13	pPeak	no	21.52 (4.37)	0.017	45	RMV	no	5.49 (1.31)	0.037
		yes	23.12 (3.79)				yes	5.88 (0.66)	
14	pPeak	no	22.65 (2.29)	0.021	46	RMV	no	5.44 (1.35)	0.031
		yes	24.00 (2.06)				yes	6.07 (0.92)	
15	pPeak	no	22.43 (2.31)	0.003	47	RMV	no	5.52 (1.29)	0.036
		yes	24.06 (1.68)				yes	5.99 (0.78)	
17	pPeak	no	22.36 (2.87)	0.007	49	RMV	no	5.50 (1.28)	0.042
		yes	24.13 (1.93)				yes	5.92 (0.64)	
18	pPeak	no	21.68 (3.81)	0.012	50	RMV	no	5.60 (1.36)	0.023
		yes	23.88 (1.97)				yes	6.05 (0.57)	
20	pPeak	no	22.41 (2.52)	0.014					
		yes	24.60 (2.53)		8	EtCO2	no	39.08 (6.18)	0.008
21	pPeak	no	22.27 (2.47)	0.02			Yes	32.27 (7.07)	
		yes	23.88 (1.89)		27	EtCO2	no	42.18 (5.76)	0.002
22	pPeak	no	22.10 (2.64)	0.06			yes	36.27 (13.26)	
		yes	23.81 (1.76)		38	EtCO2	no	44.40 (4.06)	0.043
23	pPeak	no	22.38 (2.09)	0.049			yes	40.11 (4.11)	
		yes	23.81 (2.04)		40	EtCO2	no	45.22 (4.06)	0.019
32	pPeak	no	21.41 (2.85)	0.026			yes	40.11 (3.89)	
		yes	23.81 (2.04)		41	EtCO2	no	45.33 (4.00)	0.024
37	pPeak	no	21.65 (2.15)	0.038			yes	40.78 (4.55)	
		yes	23.36 (1.91)		44	EtCO2	no	46.00 (4.42)	0.045
							yes	41.44 (3.91)	
3	FiO2	no	0.27 (0.04)	0.047	45	EtCO2	no	46.78 (4.24)	0.046
		yes	0.37 (0.62)				yes	41.89 (4.57)	
41	FiO2	no	0.26 (0.35)	0.048	46	EtCO2	no	45.67 (2.92)	0.04
		yes	0.23 (0.28)				yes	40.44 (5.64)	
					47	EtCO2	no	45.89 (2.89)	0.033
							yes	43.14 (3.72)	
					56	EtCO2	no	44.00 (3.22)	0.05
							yes	39.00 (2.65)	
					55	RF	no	9.69 (1.38)	0.042
							yes	11.38 (1.92)	

Basal ganglia injury									
Hours	Parameter	Basal ganglia injury	Mean (SD)	p	Hours	Parameter	Basal ganglia injury	Mean (SD)	p
6	EtCO2	no	42.10 (6.71)	0.025	47	EtCO2	no	46.67 (2.65)	0.004
		yes	34.76 (7.51)				yes	42.14 (2.67)	
8	EtCO2	no	38.82 (8.11)	0.027	48	EtCO2	no	46.10 (2.72)	0.021
		yes	33.24 (6.20)				yes	42.14 (3.02)	
9	ETCO2	no	41.90 (5.55)	0.007					
		yes	34.29 (7.70)		1	pPeak	no	15.56 (1.15)	0.013
16	EtCO2	no	41.90 (5.24)	0.032			yes	17.09 (2.22)	
		yes	35.36 (11.44)		6	pPeak	no	13.58 (2.34)	0.034
18	EtCO2	no	44.40 (6.20)	0.028			yes	15.57 (3.91)	
		yes	37.67 (13.66)		7	pPeak	no	15.35 (4.42)	0.006
20	EtCO2	no	45.30 (6.06)	0.037			yes	19.33 (5.43)	
		yes	37.13 (12.77)		10	pPeak	no	22.80 (3.16)	0.03
21	EtCO2	no	45.50 (6.47)	0.021			yes	23.62 (1.93)	
		yes	36.20 (12.27)		11	pPeak	no	22.84 (2.39)	0.041
24	EtCO2	no	43.90 (2.85)	0.009			yes	23.83 (1.86)	
		yes	35.00 (12.37)		12	pPeak	no	22.16 (1.43)	0.015
25	EtCO2	no	44.00 (2.58)	0.004			yes	23.45 (2.78)	
		yes	34.33 (11.81)		13	pPeak	no	21.21 (4.54)	0.025
26	EtCO2	no	43.89 (4.37)	0.002			yes	23.10 (3.66)	
		yes	34.25 (11.67)		14	pPeak	no	22.47 (1.87)	0.033
27	EtCO2	no	44.70 (3.92)	0.002			yes	23.90 (2.43)	
		yes	34.67 (12.04)		15	pPeak	no	22.21 (1.72)	0.011
28	EtCO2	no	45.00 (3.62)	0.004			yes	23.95 (2.29)	
		yes	35.25 (12.45)		17	pPeak	no	22.06 (2.31)	0.017
29	EtCO2	no	44.80 (3.71)	0.009			yes	24.05 (2.61)	
		yes	35.75 (12.68)		18	pPeak	no	44.40 (6.20)	0.027
30	EtCO2	no	47.00 (3.68)	0.02			yes	37.67 (13.66)	
		yes	36.91 (13.89)		20	pPeak	no	22.22 (2.32)	0.024
32	EtCO2	no	44.70 (2.16)	0.004			yes	24.32 (2.73)	
		yes	36.64 (12.69)		21	pPeak	no	22.11 (2.29)	0.038
33	EtCO2	no	45.60 (2.63)	0.015			yes	23.70 (2.20)	
		yes	45.20 (18.26)		22	pPeak	no	21.94 (2.18)	0.023
34	EtCO2	no	45.30 (3.37)	0.033			yes	23.68 (2.41)	
		yes	40.70 (4.45)		24	pPeak	no	22.22 (1.99)	0.045
35	EtCO2	no	45.67 (4.44)	0.044			yes	23.83 (2.46)	
		yes	41.30 (6.15)		25	pPeak	no	22.17 (1.95)	0.023
36	EtCO2	no	45.60 (2.01)	0.007			yes	24.11 (2.63)	
		yes	39.89 (3.79)		30	pPeak	no	21.94 (2.05)	0.047
37	EtCO2	no	45.50 (3.17)	0.019			yes	23.53 (2.42)	
		yes	41.44 (7.44)		37	pPeak	no	21.56 (2.16)	0.03
38	EtCO2	no	45.40 (3.17)	0.002			yes	23.33 (1.88)	
		yes	39.00 (3.28)						
39	EtCO2	no	45.00 (3.13)	0.004	1	RMV	no	4.59 (0.71)	0.009
		yes	38.89 (3.66)				yes	5.28 (0.81)	
40	EtCO2	no	45.10 (4.23)	0.012	38	RMV	no	5.56 (1.29)	0.048
		yes	39.63 (3.29)				yes	6.22 (1.13)	
41	EtCO2	no	45.00 (4.81)	0.032	40	RMV	no	5.46 (1.33)	0.034
		yes	40.63 (3.67)				yes	5.94 (0.67)	
42	EtCO2	no	45.40 (4.40)	0.023					
		yes	41.50 (2.83)		1	SpO2	no	98.13 (1.41)	0.038
43	EtCO2	no	44.20 (4.10)	0.049			yes	96.41 (2.68)	
		yes	41.50 (2.20)						
44	EtCO2	no	45.60 (5.32)	0.044	7	PEEP	no	1.67 (3.84)	0.04
		yes	41.38 (2.33)					4.81 (5.17)	
46	EtCO2	no	44.60 (5.58)	0.049					
		yes	41.13 (3.98)						

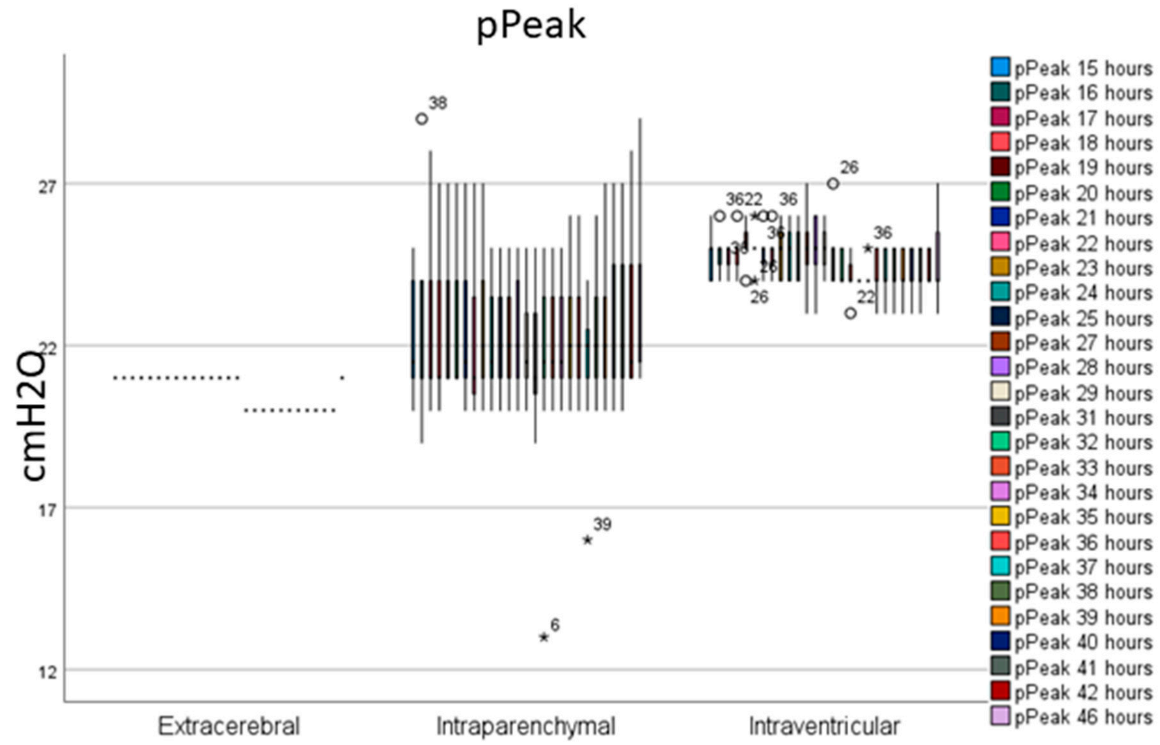

pPeak
cmH2O
27
22
17
12
Extracerebral
Intraparenchymal
Intraventricular
pPeak 15 hours
pPeak 16 hours
pPeak 17 hours
pPeak 18 hours
pPeak 19 hours
pPeak 20 hours
pPeak 21 hours
pPeak 22 hours
pPeak 23 hours
pPeak 24 hours
pPeak 25 hours
pPeak 27 hours
pPeak 28 hours
pPeak 29 hours
pPeak 31 hours
pPeak 32 hours
pPeak 33 hours
pPeak 34 hours
pPeak 35 hours
pPeak 36 hours
pPeak 37 hours
pPeak 38 hours
pPeak 39 hours
pPeak 40 hours
pPeak 41 hours
pPeak 42 hours
pPeak 46 hours

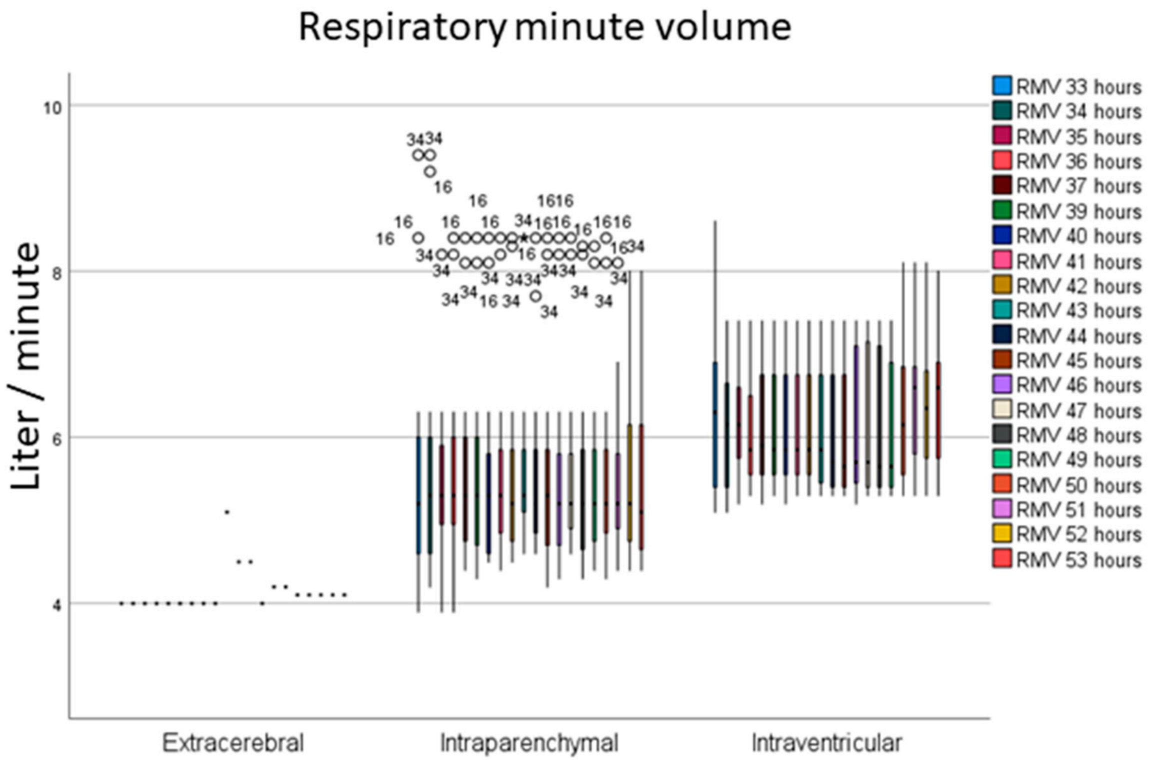

Respiratory minute volume
Liter / minute
10
8
6
4
Extracerebral
Intraparenchymal
Intraventricular
RMV 33 hours
RMV 34 hours
RMV 35 hours
RMV 36 hours
RMV 37 hours
RMV 39 hours
RMV 40 hours
RMV 41 hours
RMV 42 hours
RMV 43 hours
RMV 44 hours
RMV 45 hours
RMV 46 hours
RMV 47 hours
RMV 48 hours
RMV 49 hours
RMV 50 hours
RMV 51 hours
RMV 52 hours
RMV 53 hours
