# Supplementary material for: Unravelling Secondary Brain Injury: Insights from a Human-Sized Porcine Model of Acute Subdural Haematoma
Source: Cells. 2024 Dec 27;14(1):17. doi: 10.3390/cells14010017 (PMC11720468; doi:10.3390/cells14010017)
Supplement: Supplementary file 1 [file cells-14-00017-s001.zip › Supplement Table S4 Rev 2.pdf]

**Injury pattern** (EC = extracerebral, IP = intraparenchymal, IV = intraventricular)									
Hours	Parameter	Injury pattern	Mean (SD)	p	Hours	Parameter	Injury pattern	Mean (SD)	P
1	cLactate	EC	1.35 (0.39)*	0.023	1	pCO2	EC	37.83 (3.51)*	0.045
		IP	2.61 (0.98)*				IP	36.89 (4.21)*	
		IV	1.89 (0.78)				IV	36.95 (5.61)	
26	pH	EC	7.45 (0.08)*	0.043	26	p50e	EC	26.24 (2.64)*	0.03
		IP	7.50 (0.05)				IP	24.17 (1.73)	
		IV	7.54 (0.02)*				IV	22.91 (0.46)*	
38	cNa	EC	146.67 (1.16)	0.018	56	FMetHb	EC	1.10 (0)*	0.05
		IP	142.80 (3.95)*				IP	0.77 (0.08)*	
		IV	149.00 (1.0)*				IV	0.95 (0.88)	
44	cNa	EC	145.50 (0.58)	0.023					
		IP	142.13 (3.93)*						
		IV	147.40 (3.13)*						
**Brainstem injury**									
Hours	Parameter	Brainstem injury	Mean (SD)	p	Hours	Parameter	Brainstem injury	Mean (SD)	P
0	cK	no	3.08 (0.20)	0.026	3	pH	no	7.52 (0.04)	0.004
		yes	3.24 (0.26)				yes	7.48 (0.04)	
3	cK	no	3.02 (0.18)	0.001	26	pH	no	7.49 (0.06)	0.03
		yes	3.29 (0.26)				yes	7.53 (0.03)	
8	cK	no	3.06 (0.40)	0.027	3	ctO2c	no	13.02 (1.57)	0.026
		yes	3.37 (0.45)				yes	12.04 (1.55)	
3	Hctc	no	29.83 (3.50)	0.039	14	ctO2c	no	16.97 (2.33)	0.015
		yes	27.89 (3.26)				yes	14.12 (3.27)	
26	Hctc	no	34.33 (5.99)	0.05	32	ctO2c	no	13.65 (2.73)	0.045
		yes	28.98 (6.75)				yes	11.46 (2.65)	
3	ctHb	no	9.56 (1.07)	0.045	56	ctO2c	no	12.67 (2.30)	0.033
		yes	8.98 (1.09)				yes	8.34 (1.12)	
26	ctHb	no	11.14 (1.97)	0.043	3	p50e	no	24.56 (2.59)	0.023
		yes	9.34 (2.23)				yes	26.78 (2.99)	
3	pCO2	no	36.32 (3.85)	0.011	26	p50e	no	24.52 (1.98)	0.02
		yes	39.03 (2.62)				yes	23.06 (.074)	
14	cCa2	no	0.82 (0.19)	0.022	0	FHHb	no	0.27 (0.34)	0.03
		yes	0.70 (0.19)				yes	0.57 (0.42)	
38	cCa2	no	0.76 (0.15)	0.021					
		yes	0.61 (0.10)						
**Basal ganglia injury**									
Hours	Parameter	Basal ganglia injury	Mean (SD)	p	Hours	Parameter	Basal ganglia injury	Mean (SD)	p
0	pO2	no	163.92 (9.99)	0.01	1	cGlu	no	89.00 (13.06)	0.031
		yes	151.48 (14.60)				yes	79.32 (12.33)	
3	pO2	no	103.11 (18.42)	0.038	3	cGlu	no	99.05 (21.11)	0.021
		yes	91.33 (23.40)				yes	85.92 (18.92)	
56	pO2	no	109.47 (20.60)	0.033	32	cGlu	no	90.94 (24.98)	0.008
		yes	138.00 (6.25)				yes	78.92 (23.13)	
3	ctHb	no	9.86 (0.96)	0.001	3	Hctc	no	30.83 (3.22)	0.001
		yes	8.93 (1.04)				yes	27.70 (3.14)	
56	ctHb	no	9.17 (1.83)	0.033	44	Hctc	no	30.81 (6.27)	0.042
		yes	5.90 (0.92)				yes	25.58 (5.68)	
3	cNa	no	144.00 (1.89)	0.045	56	Hctc	no	28.46 (5.59)	0.033
		yes	142.79 (2.02)				yes	18.63 (2.73)	
8	cNa	no	144.15 (2.62)	0.009	8	pCO2	no	37.99 (4.95)	0.017
		yes	141.96 (2.33)				yes	34.61 (3.64)	
3	cK	no	2.96 (0.15)	0.001	20	pCO2	no	36.72 (2.79)	0.04
		yes	3.24 (0.26)				yes	34.76 (2.97)	
8	cK	no	2.95 (0.34)	0.002	3	ctO2	no	13.49 (1.40)	0.001
		yes	3.36 (0.43)				yes	12.01 (1.50)	
0	FO2Hb	no	98.99 (0.40)	0.013					
		yes	98.76 (0.38)						
