# Supplementary material for: Unravelling Secondary Brain Injury: Insights from a Human-Sized Porcine Model of Acute Subdural Haematoma
Source: Cells. 2024 Dec 27;14(1):17. doi: 10.3390/cells14010017 (PMC11720468; doi:10.3390/cells14010017)
Supplement: Supplementary file 1 [file cells-14-00017-s001.zip › Supplement Table S5 Rev 2.pdf]

Suppleent Table S5

			Hours						
**Parameter**	**Group**	**1**	**3**	**8**	**14**	**26**	**38**	**50**	**p**
	**Blood gas analysis**								
**pH-Value (art Base Excess)**	Extracerebral	7.5 (0.1)	7.5 (0.6)	7.5 (0.1)	7.4 (0.2)	7.5 (0.1)*	7.5 (0.1)	7.5 (0.1)	0.043
	Intraparenchymal	7.5 (0.1)	7.5 (0.1)	7.5 (0.1)	7.4 (0.1)	7.5 (0.1)*	7.5 (0.1)	7.5 (0.1)	
	Intraventricular	7.5 (0.1)	7.5 (0.1)	7.5 (0.1)	7.5 (0.2)	7.5 (0.1)*	7.5 (0.1)		
**Arterial pCO_2 [mmHg]**	Extracerebral	37.8 (3.5)*	36.6 (5.7)	38.3 (6.6)	35.7 (2.9)	37.0 (3.1)	38.3 (2.8)	40.8 (3.0)	0.045
	Intraparenchymal	36.9 (4.2)*	36.5 (3.7)	36.4 (5.0)	35.9 (4.0)	35.5 (2.5)	35.7 (2.3)	37.1 (3.2)	
	Intraventricular	37.0 (5.6)*	39.1 (2.3)	34.9 (2.4)	35.7 (2.6)	35.3 (1.2)	36.8 (0.7)		
**Arterial pO_2 [mmHg]**	Extracerebral	123.9 (37.0)	94.2 (20.7)	132.7 (54.9)	151.0 (56.6)	148.2 (61.3)	109.4 (26.1)	95.8 (1.8)	
	Intraparenchymal	129.3 (33.4)	101.1 (24.1)	180.5 (107.0)	174.3 (98.3)	158.2 (59.4)	96.8 (11.3)	99.7 (18.8)	
	Intraventricular	134.5 (29.7)	89.6 (16.8)	171.3 (136.2)	189.5 (150.1)	154.3 (57.0)	109.8 (26.5)		
**ctHb [g x dL^{-1}]**	Extracerebral	9.5 (0.9)	9.7 (1.3)	10.8 (0.8)	12.3 (2.0)	12.5 (1.3)	8.9 (1.9)	9.9 (1)	
	Intraparenchymal	9.8 (1.0)	9.5 (1.2)	11.8 (2.2)	11.8 (1.7)	10.7 (2.1)	9.8 (2.1)	9.5 (2)	
	Intraventricular	9.0 (1.3)	9.0 (0.9)	10.8 (2.3)	10.4 (3.1)	9.5 (2.3)	8.7 (1.7)		
**Hctc [% Vol.]**	Extracerebral	29.4 (2.8)	30.1 (3.9)	33.3 (2.3)	37.8 (6.1)	38.7 (3.9)	27.7 (5.8)	30.5 (3.3)	
	Intraparenchymal	30.4 (3.1)	29.4 (3.5)	35.2 (8.5)	36.4 (5.3)	33.1 (6.3)	30.2 (6.4)	29.5 (7.1)	
	Intraventricular	28.0 (3.8)	28.4 (3.6)	33.4 (6.9)	32.1 (9.2)	29.4 (7.0)	27.5 (4.8)		
**sO_2 [% Vol.]**	Extracerebral	98.7 (2.0)	96.7 (3.5)	98.6 (1.2)	99.0 (1.1)	98.7 (1.9)	98.6 (1.3)	97.6 (0)	
	Intraparenchymal	98.8 (1.3)	97.1 (2.3)	98.7 (2.6)	98.8 (2.1)	99.0 (1.5)	97.7 (1.1)	97.9	
	Intraventricular	99.1 (01.0)	95.6 (3.9)	98.9 (1.2)	98.8 (1.7)	99.3 (1.0)	98.4 (1.2)		
**FO_2Hb [% Vol.]**	Extracerebral	97.7 (1.9)	95.6 (3.4)	97.5 (1.5)	97.8 (1.5)	97.6 (2.2)	97.4 (1.6)	96.3 (0.1)	
	Intraparenchymal	97.9 (1.5)	96.0 (2.4)	97.6 (2.6)	97.7 (2.2)	97.8 (1.7)	96.3 (1.2)	96.6 (1)	
	Intraventricular	98.2 (1.0)	94.7 (3.8)	98.0 (1.3)	98.0 (1.9)	98.3 (1.2)	97.2 (1.3)		
**FCOHb [% Vol.]**	Extracerebral	0.3 (0.1)	0.3 (0.2)	0.3 (0.3)	0.3 (0.3)	0.2 (0.2)	0.5 (0.3)	0.6 (0.1)	
	Intraparenchymal	0.2 (0.2)	0.3 (0.2)	0.2 (0.2)	0.3 (0.3)	0.4 (0.2)	0.7 (0.3)	0.6 (0.3)	
	Intraventricular	0.2 (0.3)	0.7 (0.2)	0.2 (0.3)	0.3 (0.3)	0.3 (0.6)	0.5 (0.4)		
**FHHb [% Vol.]**	Extracerebral	1.3 (2.0)	3.3 (3.4)	1.4 (1.2)	1.0 (1.1)	1.3 (1.9)	1.4 (1.3)	2.4 (0)	
	Intraparenchymal	1.2 (1.3)	2.9 (2.4)	1.3 (2.5)	1.2 (2.1)	1.0 (1.5)	2.1 (1.2)	2.0 (1.2)	
	Intraventricular	0.9 (1.0)	4.5 (4.0)	1.1 (1.2)	1.1 (1.6)	0.7 (1.0)	1.6 (1.2)		
**FMetHb [% Vol.]**	Extracerebral	0.7 (0.1)	0.7 (0.2)	0.8 (0.2)	1.0 (0.4)	0.8 (0.4)	0.7 (0.1)	0.8 (0.2)	
	Intraparenchymal	0.6 (0.2)	0.7 (0.3)	0.8 (0.1)	1.0 (0.3)	0.9 (0.3)	0.8 (0.3)	0.7 (0.3)	
	Intraventricular	0.7 (0.3)	0.7 (0.3)	0.7 (0.2)	0.9 (0.6)	0.7 (0.1)	0.7 (0.3)		
**cK^+**	Extracerebral	3.0 (0.2)	2.9 (0.2)	3.0 (0.4)	3.5 (0.4)	3.5 (0.5)	3.1 (0.3)	3.1 (0.6)	

Suppleent Table S5

**(Potassium) [mmol/L]**	Intraparenchymal	3.0 (0.2)	3.1 (0.2)	3.1 (0.4)	3.7 (0.7)	3.5 (0.7)	3.3 (0.6)	3.2 (0.6)	
	Intraventricular	3.0 (0.2)	3.2 (0.3)	3.4(0.5)	3.7 (0.6)	3.2 (0.3)	3.2 (0.2)		
**cNa^+ (Sodium) [mmol/L]**	Extracerebral	139.5 (3.8)	143.0 (1.7)	143.2 (1.6)	145.5 (1.7)	144.7 (7.1)	146.7 (1.2)*	143.0 (1.4)	0.018
	Intraparenchymal	143.0 (2.9)	143.8 (1.8)	143.2 (2.8)	143.8 (3.2)	143.8 (3.7)	142.8 (4.0)*	142.9 (3.6)	
	Intraventricular	142.1 (1.8)	142.6 (2.4)	142.4 (2.8)	143.1 (2.6)	146.3 (4.6)	149.0 (1.0)*		
**Arterial cCa^{2+} [mmol/L]**	Extracerebral	0.9 (0.1)	0.8 (0.2)	0.8 (0.3)	0.7 (0.1)	0.7 (0.1)	0.7 (0.1)	0.6 (0.1)	
	Intraparenchymal	0.9 (0.2)	0.8 (0.2)	0.8 (0.2)	0.8 (0.2)	0.7 (0.2)	0.7 (0.2)	0.7 (0.2)	
	Intraventricular	0.9 (0.2)	0.9 (0.2)	0.8 (0.2)	0.7 (0.2)	0.6 (01)	0.7 (0.1)		
**Arterial cGlucose [mg x dL^{-1}]**	Extracerebral	87.8 (16.4)	103.4 (31.8)	120.2 (11.7)	119.8 (25.0)	135.7 (89.5)	72.7 (5.5)	59.0 (18.4)	
	Intraparenchymal	81.8 (12.6)	89.1 (13.6)	144.2 (51.5)	116.9 (55.3)	90.4 (30.9)	87.2 (24.6)	75.1 (24.0)	
	Intraventricular	84.6 (14.4)	92.7 (26.6)	126.1 (43.5)	108.0 (42.3)	81.4 (15.3)	92.3 (11.1)		
**cLactate [mg x dL^{-1}]**	Extracerebral	1.4 (0.4)*	1.6 (0.4)	2.6 (1.8)	5.7 (7.6)	3.3 (3.9)	0.6 (0.3)	0.7 (0)	0.023
	Intraparenchymal	2.6 (1.0)*	2.5 (0.9)	4.8 (2.4)	5.7 (4.7)	2.2 (1.8)	1.5 (1.4)	1.0 (0.5)	
	Intraventricular	1.9 (0.8)*	1.9 (1.0)	3.6 (2.3)	3.8 (5.0)	1.5 (1.2)	1.3 (0.9)		
**ctO_2 [% Vol.]**	Extracerebral	13.2 (1.1)	13.3 (2.1)	15.0 (0.9)	17.2 (2.6)	17.3 (1.5)	12.4 (2.3)	13.5 (1.4)	
	Intraparenchymal	13.7 (1.3)	12.9 (1.6)	16.6 (3.1)	16.6 (2.5)	14.7 (2.4)	12.2 (4.3)	11.8 (4.2)	
	Intraventricular	12.9 (1.7)	12.1 (1.4)	15.2 (3.5)	13.8 (3.7)	13.4 (3.1)	12.3 (2.0)		
**p50e [mmHg]**	Extracerebral	23.8 (2.1)	24.2 (3.1)	25.0 (2.9)	26.0 (5.7)	26.2 (2.6)*	23.3 (0.3)	24.1 (0.1)	
	Intraparenchymal	24.5 (2.2)	25.1 (2.6)	26.0 (2.9)	26.6 (4.5)	24.2 (1.7)*	22.0 (6.4)	22.0 (6.4)	
	Intraventricular	23.6 (1.1)	26.4 (3.3)	24.4 (2.1)	24.4 (2.1)	22.9 (0.5)*	23.2 (0.7)		
**Arterial cBase (Ecf) [mmol/L]**	Extracerebral	8.2 (3.1)	7.1 (2.9)	4.5 (4.5)	0.7 (11.1)	1.8 (6.4)	8.2 (2.7)	7.6 (2.1)	
	Intraparenchymal	5.3 (2.2)	5.2 (1.9)	1.1 (4.3)	0.2 (8.1)	4.3 (4.2)	6.7 (3.4)	7.5 (4.6)	
	Intraventricular	5.8 (3.0)	5.8 (3.0)	3.5 (5.1)	2.6 (8.1)	7.1 (2.3)	7.3 (1.6)		
**$cHCO_{3-}$ (Pst) [mmol/L]**	Extracerebral	32.1 (2.9)	31.2 (2.9)	28.7 (4.3)	25.8 (9.5)	26.4 (5.6)	32.1 (2.5)	31.3 (1.8)	
	Intraparenchymal	29.4 (2.1)	29.3 (1.8)	25.7 (3.8)	25.3 (6.9)	28.6 (3.7)	30.8 (3.1)	31.5 (4.3)	
	Intraventricular	29.9 (2.7)	29.7 (2.9)	28.0 (4.6)	26.0 (10.1)	31.2 (2.2)	31.3 (1.7)		
